# Determining 5HT_7_R’s Involvement in Modifying the Antihyperalgesic Effects of Electroacupuncture on Rats With Recurrent Migraine

**DOI:** 10.3389/fnins.2021.668616

**Published:** 2021-06-07

**Authors:** Lu Liu, Xiao-Bai Xu, Zheng-Yang Qu, Luo-Peng Zhao, Claire-Shuiqing Zhang, Zhi-Juan Li, Tian-Li Lyu, Xue-Fei Wang, Xiang-Hong Jing, Bin Li

**Affiliations:** ^1^Department of Acupuncture and Moxibustion, Beijing Hospital of Traditional Chinese Medicine, Capital Medical University, Beijing Key Laboratory of Acupuncture Neuromodulation, Beijing, China; ^2^Institute of Acupuncture and Moxibustion, China Academy of Chinese Medical Sciences, Beijing, China; ^3^Beijing Hospital of Traditional Chinese Medicine, Capital Medical University, Beijing Institute of Traditional Chinese Medicine, Beijing, China; ^4^School of Health and Biomedical Sciences, RMIT University, Melbourne, VIC, Australia

**Keywords:** 5-HT_7_ receptor, electroacupuncture, migraine pain, protein kinase A, extracellular signal-regulated kinase_1__/__2_

## Abstract

Electroacupuncture (EA) is widely used in clinical practice to relieve migraine pain. 5-HT_7_ receptor (5-HT_7_R) has been reported to play an excitatory role in neuronal systems and regulate hyperalgesic pain and neurogenic inflammation. 5-HT_7_R could influence phosphorylation of protein kinase A (PKA)- or extracellular signal-regulated kinase_1__/__2_ (ERK_1__/__2_)-mediated signaling pathways, which mediate sensitization of nociceptive neurons via interacting with cyclic adenosine monophosphate (cAMP). In this study, we evaluated the role of 5-HT_7_R in the antihyperalgesic effects of EA and the underlying mechanism through regulation of PKA and ERK_1__/__2_ in trigeminal ganglion (TG) and trigeminal nucleus caudalis (TNC). Hyperalgesia was induced in rats with dural injection of inflammatory soup (IS) to cause meningeal neurogenic inflammatory pain. Electroacupuncture was applied for 15 min every other day before IS injection. Von Frey filaments, tail-flick, hot-plate, and cold-plated tests were used to evaluate the mechanical and thermal hyperalgesia. Neuronal hyperexcitability in TNC was studied by an electrophysiological technique. The 5-HT_7_R antagonist (SB269970) or 5-HT_7_R agonist (AS19) was administered intrathecally before each IS application at 2-day intervals during the 7-day injection protocol. The changes in 5-HT_7_R and 5-HT_7_R-associated signaling pathway were examined by real-time polymerase chain reaction (RT-PCR), Western blot, immunofluorescence, and enzyme-linked immunosorbent assay (ELISA) analyses. When compared with IS group, mechanical and thermal pain thresholds of the IS + EA group were significantly increased. Furthermore, EA prevented the enhancement of both spontaneous activity and evoked responses of second-order trigeminovascular neurons in TNC. Remarkable decreases in 5-HT_7_R mRNA expression and protein levels were detected in the IS + EA group. More importantly, 5-HT_7_R agonist AS19 impaired the antihyperalgesic effects of EA on p-PKA and p-ERK_1__/__2_. Injecting 5-HT_7_R antagonist SB-269970 into the intrathecal space of IS rats mimicked the effects of EA antihyperalgesia and inhibited p-PKA and p-ERK_1__/__2_. Our findings indicate that 5-HT_7_R mediates the antihyperalgesic effects of EA on IS-induced migraine pain by regulating PKA and ERK_1__/__2_ in TG and TNC.

## Introduction

The pain associated with migraine is a major cause of its accompanying disability and can impact almost every aspect of daily living (Agosti, 2018; Collaborators, 2018). A widel-accepted mechanism posits that migraine pain is caused by activation of the trigeminovascular system (Noseda and Burstein, 2013; Akerman et al., 2017; Ashina et al., 2019), which involves algogenic and inflammatory substances such as nitric oxide, calcitonin gene-related peptide (CGRP), neurokinin A, substance P, prostaglandins, and cytokines in the meninges, whereby their release would influence the activation of trigeminovascular afferents (Goadsby and Edvinsson, 1993; Gallai et al., 1995; Sarchielli et al., 2000, 2006). These substances can alter the trigeminal nociceptor excitability through transcriptional and/or posttranslational mechanisms, giving rise to trigeminal ganglion (TG) and trigeminal nucleus caudalis (TNC) neuronal hyperexcitability. Dural application of inflammatory substances, such as capsaicin (Iwashita et al., 2013), cytokines (Zhang et al., 2012), complete Freund adjuvant (Lukács et al., 2017), or inflammatory soup (IS) (Boyer et al., 2017) are used to develop meningeal neurogenic inflammatory pain models in rodents. The sterile inflammatory phenotype is induced by the substances from trigeminal innervation, which is also known as neurogenic inflammation, and the release of these substances is associated with vasodilation, plasma extravasation secondary to capillary leakage, edema, and mast cell degranulation. Moreover, the neuroinflammatory state results in a sensitization of the terminal yielding, which provokes ongoing afferent traffic and facilitates the sensation of the peripheral terminal to the mechanical stimulation related to the local vascular pulsations (Levy, 2012; Ramachandran, 2018; Cetinkaya et al., 2020). Injection of inflammatory substances promotes phosphorylation of protein kinase A (PKA) and extracellular signal-regulated kinase_1__/__2_ (ERK_1__/__2_), which leads to the nuclear translocation and activation of cyclic adenosine monophosphate (cAMP) responsive element-binding protein (CREB) by phosphorylation at Ser133 (Levy and Strassman, 2002; Vermeer et al., 2014; Ramachandran et al., 2018). An increase in p-CREB allows for more transcriptional regulation of neuronal activation marker c-Fos resulting in the maintenance of facilitated trigeminovascular pain transmission.

Electroacupuncture (EA) is one of the typical treatments of traditional Chinese medicine, and it is widely used in clinical practice to relieve migraine pain (Li et al., 2012; Yang et al., 2014; Linde et al., 2016; Zhao et al., 2017). p-PKA and p-ERK are considered to be the key to activate nociceptive neurons and maintain peripheral and central sensitization (Giniatullin et al., 2008; Iwashita et al., 2013; Nakaya et al., 2016). Our previous investigation found EA shows a direct effect on nociceptive neurons (Qu et al., 2020a, b). In addition, several studies (Han et al., 2015; Shao et al., 2016; Hu et al., 2020) suggested PKA and ERK_1__/__2_ in the spinal dorsal horn might involve EA antihyperalgesia. However, the participation of PKA and ERK_1__/__2_ in EA antihyperalgesia has not been well defined.

5-Hydroxytryptamine (5-HT), a biogenic amine, is associated with feeding behavior, thermoregulation, sexual behavior, and sleep (Silberstein, 1994). The association between 5-HT and migraine pathophysiology has occurred ever since the observation of Sicuteri that the urinary excretion of 5-HT metabolites increased during migraine attacks (Sicuteri et al., 1961). The most convincing change of 5-HT metabolism during migraine attack could be the low central 5-HT disposition together with the increase in 5-HT release (Panconesi, 2008). Some lines of evidence come from drugs that interact with 5-HT receptors as migraine therapeutic targets; especially in the 1990s, the therapies targeting the 5-HT receptor produce selective cranial extracerebral vasoconstriction and inhibition of the trigeminovascular system responses (Villalón and VanDenBrink, 2017).

5-Hydroxytryptamine (5-HT)_7_ receptor (5-HT_7_R), belonging to the G protein-coupled receptor superfamily, is one of the most recently discovered members of the serotonin receptor family. Further studies showed that 5-HT_7_R is associated with mood disorder (Sarkisyan et al., 2010), epilepsy (Yang et al., 2012), autism spectrum disorder (Ciranna and Catania, 2014), nociception (Brenchat et al., 2010), and migraine (Wang et al., 2014). 5-HT_7_R are abundantly expressed in the central and peripheral nervous system, mainly in the thalamus (Varnäs et al., 2004), hypothalamus (Bonaventure et al., 2004), hippocampus, cerebral cortex (Neumaier et al., 2001), amygdala (Muneoka and Takigawa, 2003), cerebellum (Geurts et al., 2002), TNC (Martínez-García et al., 2011), and TG (Terrón et al., 2001) and have been reported to play an excitatory role in neuronal systems and regulate hyperalgesic pain and neurogenic inflammation (Terrón, 2002; Bravo et al., 2019), 5-HT_7_R couples positively to adenylate cyclase (AC) through activating Gαs (the stimulatory Gs protein), resulting in an increase in cAMP (Baker et al., 1998; Hamblin et al., 1998). Recent studies showed that 5-HT_7_R could influence PKA- or ERK_1__/__2_-mediated signaling pathways, which mediate sensitization of nociceptive neurons via interacting with Gαs-cAMP (Ohta et al., 2006; Cho et al., 2015), Previous studies found that blockade of 5-HT_7_R mediated craniovascular vasodilatation and perivascular trigeminal nerve endings in migraine rats. Furthermore, the 5-HT_7_R antagonist SB-269970 reduced neurogenic dural vasodilation (Wang et al., 2014) and CGRP release (Wang et al., 2010); CGRP is a key neuropeptide in the pathophysiology of migraine.

Given that EA could prevent the phosphorylation of PKA and ERK_1__/__2_, while the activation of 5-HT_7_R could cause PKA and ERK_1__/__2_ phosphorylation levels to increase, we hypothesized that 5-HT_7_R mediates the antihyperalgesic effects of EA by regulating PKA and ERK_1__/__2_ through Gαs-cAMP signaling.

## Materials and Methods

### Animals

Male Sprague-Dawley rats weighing 210–260 g (National Institutes for Food and Drug Control, Beijing, China, Certificate number: SYXK 2014-0013) were housed at 20–26°C in plastic cages (size: 461 mm × 274 mm × 229 mm; three to four rats per cage) on soft bedding with *ad libitum* water and food under a 12-h/12-h light/dark cycle for at least 1 week before the experiment. Every effort was made to minimize the number of animals used. Numbers of animals were selected according to previous experience, i.e., a trade-off between reaching routine sample sizes for field experiments while minimizing numbers of animals for pain experiments. Experiments were performed on 100 animals (5–10 rats/group). Rats were randomized into treatment groups before their assessment. All experiments, analysis, and reporting were compliant to the animals in research: reporting *in vivo* experiments (ARRIVE). Animal experiments were performed according to the ethical guidelines set by the International Association for the Study of Pain (Zimmermann, 1983). The protocols applied here for animal care and use were approved by the Animal Experimentation Ethics Committee of Beijing Institute of Traditional Chinese Medicine (Approval number: 2018080102).

### Dural Cannulation and Inflammatory Soup Injection

The protocols used for the dural cannulation were similar to those described previously (Boyer et al., 2017; Qu et al., 2020a). A previous study found that the effects of inflammatory soup (IS) application on the dura was due to the activation of trigeminal afferents and not to a systemic (Boyer et al., 2014). Rats were briefly anesthetized with sodium pentobarbital (40 mg kg^–1^, ip). The dura of rats was exposed through a 1-mm hole (1 mm left of midline, 1 mm anterior to bregma) and a guide cannula, extending 0.5 mm from the skull surface to avoid irritation of the dural tissue, was inserted into the hole, and secured into place with dental acrylic (see Supplementary Figure 1A). One week postsurgery, injections (20 μl, 1 μl min^–1^) of IS or artificial cerebrospinal fluid (aCSF) were performed under brief anesthesia (isoflurane, 3% induction, 1.5% maintenance) through the cannula at 2-day intervals during the 7-day injection protocol (Boyer et al., 2017; Bishop et al., 2019; see Supplementary Figure 1B). Twenty microliters of aCSF were injected as in rats which form the control (Con) group. After recovery, rats were gently placed into the glass chamber for behavioral testing. IS was formulated with histamine, serotonin, and bradykinin, all at 2 mM, and prostaglandin E2 (PGE2), at 0.2 mM, respectively, in 10 mM HEPES buffer at pH 5.0, and aCSF contained 124 mM NaCl, 2.5 mM KCl, 1.2 mM NaH_2_PO_4_, 1.0 mM MgCl_2_, 2.0 mM CaCl_2_, 25 mM NaHCO_3_, and 10 mM glucose.

### Direct Transcutaneous Intrathecal Injection

Intrathecal injection was performed as described previously (Lapirot et al., 2009). One week after dural cannulation, rats received direct transcutaneous intrathecal injection before each IS application at 2 day intervals during the 7 day injection protocol. Under isoflurane-induced anesthesia (3% induction, 1.5% maintenance), a 25-G needle connected to a 25-μl Hamilton syringe was inserted percutaneously into the vertebral canal between L5 and L6. A tail-flick reaction denoted a successful puncture. A rat was injected intrathecally with 20 μl of 1% Chicago sky blue (C8678, Sigma-Aldrich, United States) to determine whether the drug injected intrathecally was able to reach the L6-S1 spinal cord level. After that, SB269970 (5-HT_7_R antagonist, 7 μl, 5 mM dissolved in distilled water; Sigma-Aldrich, United States) or AS-19 (5-HT_7_R agonist, 15 μl, 10 μM dissolved in saline; Sigma-Aldrich, United States) was administered intrathecally with an injection rate of 1 μl min^–1^ (Hoffman and Mitchell, 2011; Ni et al., 2019). Rats that exhibit signs of motor impairment are excluded from the experiment.

### Electroacupuncture

**FIGURE 1:**
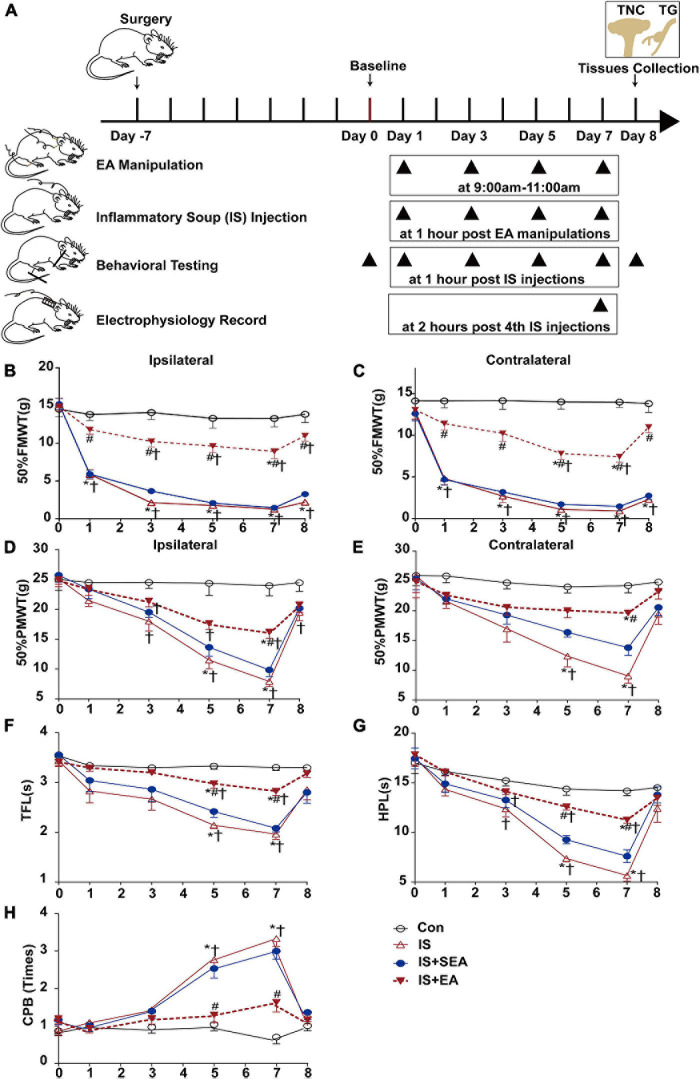
Effects of EA on IS-induced mechanical and thermal hyperalgesia. **(A)** Scheme of the experimental design. Rats were applied EA manipulation, following repeated-dural IS injection on days 1, 3, 5, and 7, then measurements of 50%FMWT, 50%PMWT, TFL, HPL, and CPB to examine the extent of mechanical and thermal hyperalgesia after 1 h of IS injection and also the day 0 without IS injection and 1 day after the 4th IS injection (day 8). **(B,C)** Reduction in 50%FMWT of IS + EA and IS compared with the Con group (*n* = 10; *P < 0.05); increase in 50%FMWT of IS + EA compared with the IS group on the bilateral side (*n* = 10; ^#^*P* < 0.05). **(D,E)** Effects of EA on extracephalic mechanical hyperalgesia after dural IS injection. There was an increase of the 50%PMWT in the IS + EA group compared with the IS group on the bilateral side (*n* = 10, ^#^*P* < 0.05). The TFL **(F)**, and HPL **(G)** decrease and CPB **(H)** increase in the IS group compared with the Con group (*n* = 10, *P < 0.05) on days 5 and 7, there was an increase of the TFL and HPL and a decrease of CPB in the IS + EA compared with the IS group (*n* = 10, ^#^*P* < 0.05). The repeated-measure two-way ANOVA *post hoc* Tukey multiple comparisons test was used. Group values are indicated by mean ± SEM. *P < 0.05 compared with the Con group; ^#^*P* < 0.05 compared with the IS group at the same time point; ^†^*P* < 0.05 compared with the baseline on day 0; 50%FMWT, 50% facial mechanical withdrawal threshold; 50%PMWT, 50% paw mechanical withdrawal threshold; TFL, tail-flick latency; HPL, hot-plate latency; CPB, cold-plate behaviors; EA, electroacupuncture; IS, inflammatory soup; SEA, sham electroacupuncture; Con, control.

Rats in GB20 + GB34, GB20 + SJ17, GB34 + ST36, IS + EA, and IS + AS19 + EA group received EA stimulation under anesthetization with isoflurane (3% induction, 1.5% maintenance). The Con, IS and IS + SEA group were also anesthetized at the same time. After immobilization, the rats were scrubbed with 75% alcohol disinfectant at the EA sites before EA was being performed by the acupuncturist with acupuncture needles. For the EA stimulation, four needles, each sized at 0.25 mm × 13 mm (Suzhou Medical Appliance Factory, Suzhou, China) were inserted at a depth of 1.5 mm in the bilateral head and hind leg at acupoints corresponding to Fengchi (GB20), Yifeng (SJ17), Yanglingquan (GB34), and Zusanli (ST36) (see Supplementary Figure 2 and Supplementary Table 1), as described previously (Zhang et al., 2016; Wang X.R. et al., 2018; Xu et al., 2019; Zhao et al., 2020). In clinical practice, GB20, SJ17, GB34, and ST36 were utilized most frequently in the clinical treatment for decreasing days of migraine attacks (Allais et al., 2002; Li et al., 2009; Wang et al., 2011; Li et al., 2012; Zhao et al., 2017). The needles were connected to HANS LH202H Han’s acupoint nerve stimulator (Beijing Hua Wei Industrial Development Co, Beijing, China). Electroacupuncture stimulation (2–15 Hz alternating wave, 1.0 mA) of 15 min was initiated before IS injection. This process was repeated every 2 days (Figure 1A). Sham electroacupuncture (SEA) was acupuncture needle insertion into non-acupoints (approximately 10 and 15 mm above the iliac crest) without electrical stimulation.

### Experimental Procedures

#### Experiment I

To find the best treatment protocol, we compared the effects of electroacupuncture at different acupoints GB20 + GB34, GB20 + SJ17, and GB34 + ST36, in changing the cutaneous hyperalgesia and 5-HT_7_R expression of IS rats. The rats were randomly divided into six groups: repeated dural injection of aCSF (Con group), repeated dural injection of IS (IS group), IS with sham EA (IS + SEA group), IS with EA at GB20 and GB34 point (GB20 + GB34 group), IS with EA at GB20 and SJ17 point (GB20 + SJ17 group), and IS with EA at GB34 and ST36 point (GB34 + ST36 group) group (*n* = 10 per group). We performed the 50% facial mechanical withdrawal threshold (50%FMWT), 50% paw mechanical withdrawal threshold (50%PMWT), tail-flick latency (TFL), hot-plate latency (HPL), and cold-plate behaviors (CPB) (Fregni et al., 2018) to evaluate the mechanical and thermal hyperalgesia. 5-HT_7_R expression in TG and TNC were detected by real-time polymerase chain reaction (RT-PCR), Western blot, and immunofluorescence analyses.

#### Experiment II

To observe the therapeutic effects of electroacupuncture, we examined whether EA can alleviate cutaneous hyperalgesia and neuronal hyperexcitability in TNC, which is induced by repeated dural injection of IS, using behavioral testing and electrophysiological recordings. The rats were assigned at random to Con group, IS group, IS + SEA group, IS + EA (best treatment protocol from experiment I) group (*n* = 10 per group).

#### Experiment III

To investigate whether electroacupuncture affects 5-HT_7_R-associated PKA and ERK_1__/__2_ phosphorylation signaling pathway, we examined 5-HT_7_R, cAMP production, the total amount of PKA, ERK_1__/__2_, and CREB, phosphorylation of PKA, ERK_1__/__2_, CREB, and c-Fos expression by RT-PCR, Western blot, immunofluorescence, and enzyme-linked immunosorbent assay (ELISA) analyses in TG and TNC. The animal groups in experiment III were the same as those in experiment II (*n* = 5 per group).

#### Experiment IV

To determine whether 5-HT_7_R-associated PKA and ERK_1__/__2_ phosphorylation signaling pathways are involved in EA’s therapeutic effects, we tested cutaneous hyperalgesia, neuronal hyperexcitability in TNC, and 5-HT_7_R-related protein using behavioral testing, electrophysiological recordings, Western blot, and ELISA analyses. The rats were randomly divided into four groups: IS group, IS + SB269970 group (IS with 5-HT_7_R antagonist SB269970), IS + AS19 + EA group (IS + EA with 5-HT_7_R agonist AS19), and IS + EA group (*n* = 5 per group).

### Behavioral Testing

Before their behavioral testing, the rats were acclimatized to the experiment’s environment by being exposed to it for 30 min a day for 2 days, and those with an abnormal baseline were excluded. To avoid experimenter bias, the investigator who conducted the behavioral study did not know the type of treatment applied to the rats. On each of the testing days, rats were brought into the testing room for 30 min to acclimate them to the environment. As the rats were acclimated to the environment, they did not show signs of anxiety such as defecation or urination, nor did they attempt to escape by climbing onto the plastic cylinder or cage. After the determination of the baseline response (day 0), all behavioral testing was assessed on days 1, 3, 5, and 7 after 1 h of IS injection, and day 8 without IS injection.

#### Mechanical Withdrawal Threshold

Von Frey filaments (Stoelting, Wood Dale, IL, United States) were used to measure the 50% withdrawal threshold using the up-and-down method reported by Dixon (1980) and Chaplan et al. (1994). Tests were conducted during the day portion of the circadian cycle only (06:00–18:00 h). For 50%FMWT testing, the rats were placed in a plastic cylinder with two rectangle open windows which allowed full access to the bilateral face. For 50%PMWT testing, the rats were placed in a plastic cylinder with a wire mesh bottom that allowed for full access to the bilateral paws. Behavioral accommodation was allowed for approximately 15 min until cage exploration and major grooming activities ceased. The areas tested were the face and the midplantar hind paw. The face and paw were touched with one of a series of 10 von Frey filaments with logarithmically incremental stiffness (0.4, 0.6, 1, 2, 4, 6, 8, 15, 26, and 60 Fg). A series of filaments, starting with one that had a buckling weight of 2 g, was applied in a consecutive sequence on the bilateral facial and hind paw with a pressure causing the filament to buckle and held for approximately 6–8 s.

The von Frey filaments were presented perpendicular to the facial and plantar surface with sufficient force to cause slight buckling against the bilateral face and paw. Stimuli were presented at intervals of 10 s, allowing for apparent resolution of any behavioral responses to previous stimuli. A positive response was noted if the face and paw were sharply withdrawn. Based on previous studies, the cut-off of a 60-g filament was selected as the upper limit for testing, since stiffer filaments tended to move the entire face or limb rather than to buckle, substantially changing the nature of the stimulus.

An optimal threshold calculation by this method requires six responses in the immediate vicinity of the 50% threshold (Dixon, 1980). Since the threshold is not known, strings of similar responses may be generated as the threshold is approached from either direction. Despite having all responses recorded, the counting of the critical 6 data points did not commence until the response threshold was first crossed, at which the time the two responses straddling the threshold were retrospectively selected as the first two responses of the series of six. Four additional responses to the continued presence of stimuli that were varied sequentially up or down, based on the rat’s response, accounted for the remainder of the series (Lopez-Canul et al., 2015).

#### Tail-Flick Latency

The tail-flick test was used to assess thermal hyperalgesia in the rats. The rats were placed in a plastic cylinder from which its tail protruded. The presence of tail flicking was considered withdrawal latency. The intensity of the light from the thermal stimulator (IITC Inc., Woodland Hills, CA, United States) was adjusted at the start of the experiment such that average baseline latencies were approximately 3 to 4 s, and a cutoff latency of 16 s was established. The heat was directed to the distal tail (20 mm from the tip). Three trials were done at intervals of 5 min, and the latency (seconds) was the average of three trials (Yadlapalli et al., 2016).

#### Hot-Plate Latency

The hot-plate test was used to assess thermal hyperalgesia in the rats. The rats were placed on a heated (50 ± 1°C) surface one at a time. Paw licking or jumping was considered withdrawal latency. A cutoff latency of 50 s was established. Three trials were done at intervals of 20 min, and the latency (seconds) was the average of three trials (Kooshki et al., 2020; Lopes et al., 2020).

#### Cold-Plate Behaviors

The cold-plate test was used to assess thermal hyperalgesia in rats. The plate was cooled down to 4 ± 1°C. Rats were then placed on the plate for 5 min and scored. Rats that were walking freely on the 4°C cold plate for 5 min did not exhibit any signs of skin injury, given that this was a mild nociceptive stimulus. The number of paw lifts quantified the response to cold during an observation period of 5 min. Three trials were done at intervals of 10 min, and the cold plate behaviors (times) were the average of three trials (Jasmin et al., 1998; Gong and Jasmin, 2017).

### Electrophysiological Recordings in the Trigeminal Nucleus Caudalis

#### Animal Preparation

The last IS injection occurred on day 7, and electrophysiological recordings were performed at 2 h postinjection. As previously described, rats were anesthetized with isoflurane (3% induction, 1.5% maintenance) mixed with oxygen. After the trachea was cannulated and the carotid artery and external jugular vein catheterized, rats were paralyzed by intravenous perfusion of vecuronium bromide (2.4 mg h^–1^) and artificially ventilated with a volume-controlled pump (54–55 strokes min^–1^). Levels of isoflurane, O_2_, N_2_, and end-tidal CO_2_ (3.5–4.5%) were measured by an anesthetic gas analyzer (Drager Vamos) during the entire experimental period. The mean arterial blood pressure was continuously monitored (90 to 110 mmHg). The colorectal temperature was kept constant at 38 ± 0.5°C. The eyes of the rat were protected with erythromycin ointment.

**FIGURE 2:**
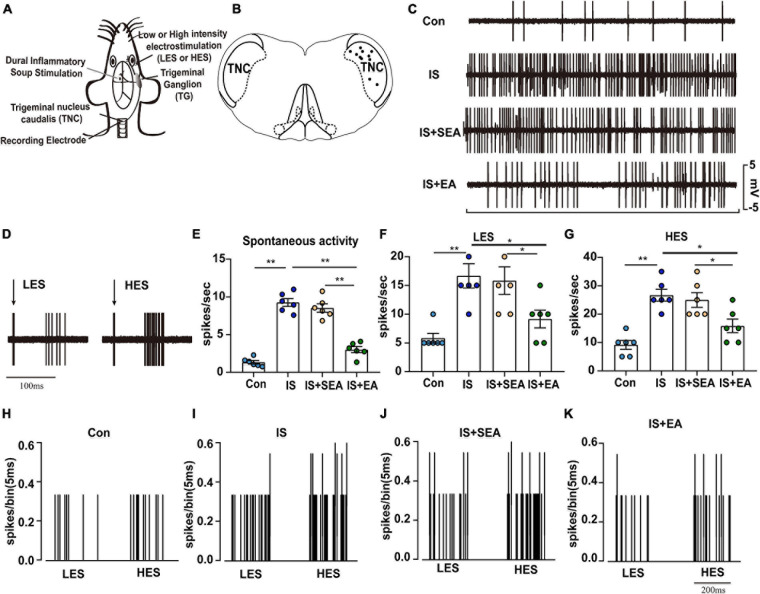
Overview of the electrophysiological recordings and neuronal characteristics among the Con, IS, IS + SEA, and IS + EA groups. **(A)** Experimental setup with IS injection, recording of neurons in the TNC and percutaneous electrical stimulation. **(B)** The location of recording sites in the TNC from which recordings of nociceptive neurons, receiving convergent input from the dura mater and facial receptive field, were made. The locations were reconstructed from lesions (∙) and located in laminae IV–VI. **(C)** Spontaneous activity was recorded for 60 s at 30 min postfinding the activated neurons for stabilization among the indicated groups. **(D)** An original tracing from a typical unit (second-order neurons) responding to percutaneous electrical stimulation in low [1 Tc (2 mA), LES] and high [2.5 Tc (5 mA), HES] intensity (latencies in the C-fiber range). Black arrow represents stimulus artifact. Spontaneous activity **(E)**, LES-evoked responses **(F)**, and HES-evoked responses **(G)** of WDR neurons recorded 2 h after the 4th IS or aCSF injection in the TCC of none manipulation (Con and IS) **(H,I)**, SEA (IS + SEA) **(J)**, or EA (IS + EA) **(K)** rats. When four groups were compared for electrophysiology, a Kruskal-Wallis test was used, and when only two groups were compared, a Mann-Whitney *U* test was used. Group values are indicated by mean ± SEM. *P < 0.05, **P < 0.01. Tc, the stimulation intensities required to evoke neuronal activity with a conductive velocity of 0.4–2 m s^–1^, namely about 2 mA. LES, low-intensity electrostimulation; HES, high-intensity electrostimulation; WDR, wide-dynamic range; TG, trigeminal ganglion; TNC, trigeminal nucleus caudalis; EA, electroacupuncture; IS, inflammatory soup; aCSF, artificial cerebrospinal fluid; SEA, sham electroacupuncture; Con, control.

The rats were placed in a stereotaxic frame with the head fixed in a ventro-flexed position. The TNC was exposed by removing the overlying musculature, atlanto-occipital membrane, dura mater, and a cervical laminectomy before being flooded with aCSF. The tungsten recording electrode with a diameter of 75 μm, tip diameter of 3–4 μm, and an impedance of 10–15 kΩ (TM33CCINS, World Precision Instruments Shanghai Trading Co., Ltd., China) was carefully implanted into a depth of 0.5–1.5 mm using a microelectrode manipulator (SR-1N, NARISHIGE Co., Ltd., Japan) (Figure 2A).

#### Electrophysiological Recording

Single-unit activities were sampled and analyzed at 300 Hz–300 kHz and were amplified 1,000 times and displayed on oscilloscopes. The activities went into a window discriminator connected to a CED 1401plus interface (Cambridge Electronic Design) and a computer (Spike 2 version 7.03a software, CED, Cambridge, United Kingdom), which allowed sampling and analysis of the spontaneous and evoked neuronal activity. Wide dynamic range (WDR) neurons were recognized based on their responses to mechanical non-noxious (brushing with a soft brush) and noxious (pinch with forceps) stimulations of their receptive fields as previously described. Specifically, each neuron that responded in a graded manner with increasing firing rates to the stimulus range from non-noxious to noxious intensity was classified as a WDR cell (see Supplementary Figure 3). Once a neuron was identified, electrical square-wave stimuli (1.5 ms duration) were applied through a pair of stainless-steel needle electrodes subcutaneously placed into the center of the receptive field; the thresholds for eliciting A- and C-fiber-evoked responses were determined. In poststimulus time histograms (PSTHs), A- and C-fiber-evoked responses were distinguished by their latencies, but only C-fiber-evoked responses were considered in the detailed analysis. Previous research showed that burst discharges at latencies ≥ 30 ms are elicited by C-fibers (Dixon, 1980; Hu, 1990). Therefore, all spikes occurring between 30 and 200 ms after a stimulus were considered to be C-fiber evoked. The C-fiber thresholds (Tc) were defined as the stimulation intensities required to evoke neuronal activity with a conductive velocity of 0.4–2 m s^–1^ (Burstein et al., 1998). Electrostimulation (ES) of 1 Tc (2 mA) and 2.5 Tc (5 mA) were applied to the facial region in this part of the experiment (Wang et al., 2020). ES comprising square waves consisted of low-intensity (2 mA, 0.75 Hz) and high-intensity (5 mA, 0.75 Hz) stimulation, and ES being applied once for 1.5 ms in order, to the most sensitive portion of the cutaneous receptive field. The responsiveness of electrical stimulation was assessed by recording the responses to 1.5 ms application of ES at 2 and 5 mA. Spontaneous activity (spikes s^–1^ for 60 s, 30 min after finding the activated neurons for stabilization) was also recorded. Only one cell was tested in each animal, and only cells with spike amplitude of more than 3 mV showing no change in amplitude or waveform throughout the experimental procedure were considered and sorted.

### Immunofluorescence

#### Tissue Processing for Immunofluorescence

Upon deep anesthesia by intraperitoneal injection of sodium pentobarbital (150 mg kg^–1^), the rat was perfused transcardially with normal saline followed by 4% paraformaldehyde in PBS for 20 min. Brain and trigeminal ganglia were isolated and postfixed for 30 min in the same fixative at room temperature. All specimens were dehydrated for cryoprotection in 30% sucrose in 0.1 M phosphate buffer (0.08 M K_2_HPO_4_, 0.02 M NaH_2_PO_4_, pH 7.4). TG and TNC in random orientation were cut with a cryostat into 20 μm sections and mounted directly onto gelatin-coated slides. From five TG and TNC sections (100 μm apart) in each rat, a total of 25 sections were selected for measurement in each group.

#### Single-Labeling Immunofluorescence

The sections obtained were washed in Tris-buffered saline (TBS, 25 mM Tris, pH 7.5) and then TBS containing 0.3% Triton X-100 (TBST), before being treated with 0.3% hydrogen peroxide in TBS to exhaust endogenous hydrogen peroxidase activity until the bubbles disappeared. Non-specific binding was blocked by 3% normal serum (from an animal species same as the secondary antibody) and 2% bovine serum albumin in TBST for 1.5 h. Sections were incubated with primary antibodies goat anti-CGRP (1:100, ab36001, Abcam, Cambridge, United Kingdom) in TBST at room temperature overnight containing 5% normal serum. After overnight reaction with the primary antibody, the sections were washed with TBST and then incubated for 1.5 h with secondary antibodies rabbit antigoat IgG labeled with FITC (1:200, RAG001, MultiSciences Biotech Co., Ltd., Hangzhou, China).

#### Double-Labeling Immunofluorescence

The following procedures were used for two primary antibodies derived from different species. Sections were processed similarly to that described under the section “single-antigen immunohistochemistry.” Primary antibodies included rabbit anti-5-HT_7_R (1:200, ab61562, Abcam, Cambridge, United Kingdom), mouse anti-NeuN (1:1,000, ab104224, Abcam, Cambridge, United Kingdom). Secondary antibodies included goat antirabbit IgG labeled with Alexa 488 (1:200, A0432, Beyotime Biotech Inc., Shanghai, China) and goat antimouse IgG labeled with Cy3 (1:200, A0432, Beyotime Biotech Inc., Shanghai, China). Sections on slides were dehydrated through an ethanol gradient/xylene and cover-slipped in an antifade mounting medium with DAPI (H-1500, Vector Laboratories, Inc., Burlingame, CA, United States). The images were captured using a Leica semiautomatic light microscope (Leica DM5500B) and processed with the Adobe Photoshop CS2 software. The CGRP-, 5-HT_7_ R-, and NeuN-positive fluorescence images were measured in squares at ×20 magnification. ImageJ 1.40 g (National Institutes of Health, Bethesda, MD, United States) was used as the software to circle the immunoreactive regions on the soma of TG and TNC neurons.

### Western Blot

ImageJ 1.40 g (National Institutes of Health, Bethesda, MD, United States) was used as the software to circle the immunoreactive regions on the soma of TG and TNC neurons. Punches of TNC regions were selected using a magnifying glass with stainless steel cannulae of 1,000 μm inner diameter and subsequently pooled. The tissue was homogenized on the ice with the lysis buffer. The supernatant was collected after centrifugation at 4°C for 20 min at 12,000 rpm. After being heated at 99°C for 5 min, the samples (20 μg/sample) were loaded onto a 5% stacking/10% separating SDS-polyacrylamide gel and then electrophoretically transferred onto a PVDF membrane (Millipore, MA, United States). The membranes were incubated first in the blocking buffer (3% non-fat milk plus 0.1% Tween-20 in the Tris-buffered saline) for 2 h at room temperature and then in the primary antibodies at 4°C overnight. The primary antibodies included the following: rabbit anti-5-HT_7_R (1:2,000, ab128892, Abcam, Cambridge, United Kingdom), mouse anti-c-Fos (1:2,000, ab190289, Abcam, Cambridge, United Kingdom), rabbit anti-PKA (1:2,000, 4782, CST, United States), rabbit anti-p-PKA (1:2,000 5661, CST, United States), rabbit anti-ERK_1__/__2_ (1:2,000, 4695, CST, United States), rabbit anti-p-ERK_1__/__2_ (1:2,000, 4370, CST, United States), rabbit anti-CREB (1:2,000, 4820, CST, United States), rabbit anti-p-CREB (1:2,000, 9198, CST, United States), and mouse anti-β-actin (1:2,000, C1313, Applygen Technologies Inc., Beijing, China). After the membranes were incubated with either the horseradish peroxidase-conjugated antirabbit or antimouse secondary antibody (1:200, 70-GAM007, MultiSciences, Biotech Co., Ltd, Hangzhou, China) at room temperature for 1 h, the enhanced chemiluminescence reagent (Bio-Rad Laboratories) was used to visualize the proteins. ChemiDoc XRS and System with Image Lab software (Bio-Rad Laboratories) were performed to generate the images. ImageJ 1.40 g software (National Institutes of Health, Bethesda, MD, United States) was used to quantify the intensity of the images. The band intensities for 5-HT_7_R and c-Fos proteins were normalized to β-actin, and those for PKA, ERK_1__/__2_, and CREB phosphorylation proteins were normalized to these total proteins.

### Enzyme-Linked Immunosorbent Assay

The endogenous levels of cAMP were measured in TG and TNC using an enzyme immunoassay kit (EXP110254, Expandbio, Beijing, China) according to the manufacturer’s instructions. The concentrations of cAMP were expressed as picomoles per milligram of protein concentrations calculated in these supernatants.

### Real-Time Polymerase Chain Reaction

Total RNA was extracted from TG and TNC with Trizol reagent (Tiangen Biotech Co., Ltd., Beijing, China). cDNA was synthesized using the PrimeScript RT reagent Kit with gDNA Eraser (RR047B, TaKaRa, Dalian, China). RT-PCR was performed in a real-time PCR system (ABI7500; Applied Biosystems, Foster City, CA, United States) using the SYBR Premix Ex Taq II (Tli RNaseH Plus) ROX plus (RR42LR, TaKaRa, Dalian, China). The PCR cycle parameters consist of an initial 30 s incubation at 95°C, followed by 40 cycles of 95°C for 5 s, 60°C for 40 s, followed by a melt from 60 to 99°C. Each primer sequence was as follows: *5-ht7r*: 5′-GGT GAG GCA AAA TGG GAA ATG-3′ (forward) and 5′-ACC GCA GTG GAG TAG ATC GTG TAG-3′ (reverse); *Gapdh*: 5′-TGG AGT CTA CTG GCG TCT T-3′ (forward) and 5′-TGT CAT ATT TCT CGT GGT TCA-3′ (reverse). The Ct value of each gene was normalized against that of *Gapdh*. Relative levels of expression were calculated using the comparative (2^–ΔΔ*Ct*^) method.

### Statistical Analyses

All statistical analyses were performed using SAS version 9.4 software. Statistical differences were determined using one-way analysis of variance for immunofluorescence, Western blot, ELISA, RT-PCR and, and two-way repeated-measures analysis of variance was applied for the data from the behavioral testing. When four groups were compared for electrophysiology, a Kruskal-Wallis test was used, and when only two groups were compared, a Mann-Whitney *U* test was used. Data in the text and figures were shown as mean ± SEM. The level of significance was set at *P* < 0.05.

## Results

### Electroacupuncture Significantly Relieved the Inflammatory Soup-Induced Hyperalgesia

**FIGURE 3:**
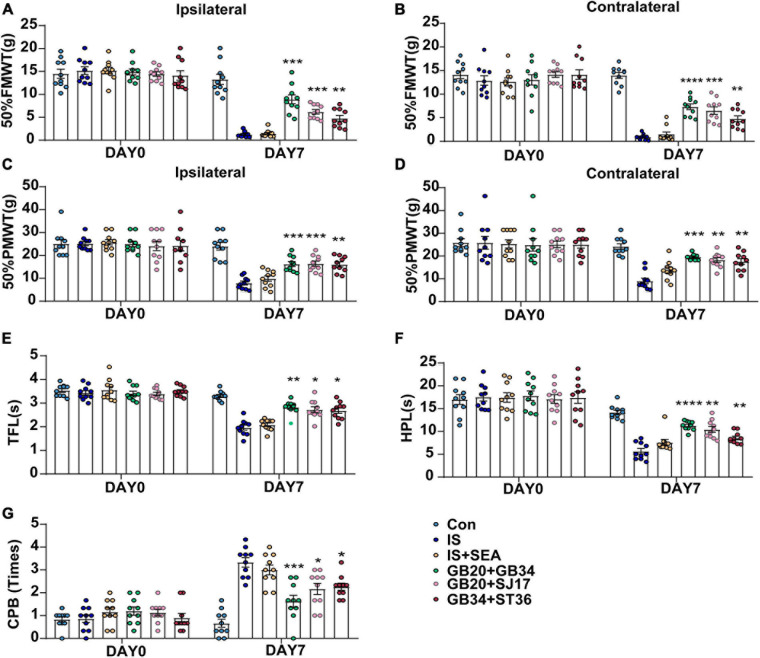
Effect of EA applied at different acupoints on day 0 without IS injection and day 8 (1 day after the 4th IS injection) among Con, IS, IS + SEA, GB20 + GB34, GB20 + SJ17, and GB34 + ST36 groups. Changes in mechanical [50% FMWT **(A,B)**, and 50% PMWT **(C,D)**] and thermal [TFL **(E)**, HPL **(F)**, and CPB **(G)**] hyperalgesia in dural aCSF- and IS-injected rats that received four times EA at GB20 + GB34, GB20 + SJ17, GB34 + ST36, or non-acupoints every other day (*n* = 10). One-way ANOVA followed by *post hoc* Tukey test. Group values are indicated by mean ± SEM. *P < 0.05, **P < 0.01, ***P < 0.001, ****P < 0.0001, compared with the IS group. 50%FMWT, 50% facial mechanical withdrawal threshold; 50%PMWT, 50% paw mechanical withdrawal threshold; TFL, tail-flick latency; HPL, hot-plate latency; CPB, cold-plate behaviors; EA, electroacupuncture; IS, inflammatory soup; aCSF, artificial cerebrospinal fluid; GB20, Fengchi; GB34, Yanglingquan; SJ17, Yifeng; ST36, Zusanli.

Measurements of 50%FMWT, 50%PMWT, TFL, HPL, and CPB were used to examine the extent of mechanical and thermal hyperalgesia induced by repeated IS infusion and the antihyperalgesic effects of EA. We compared the effects of electroacupuncture at different acupoints, including GB20 + GB34, GB20 + SJ17, and GB34 + ST36 (Figures 3A–G), in changing the mechanical and thermal hyperalgesia of IS rats to select the best treatment protocol. We found that EA at GB20 + GB34, GB20 + SJ17, or GB34 + ST36 reduced the cutaneous hyperalgesia. The GB20 + GB34 group showed the best effect that reduced cephalic mechanical (GB20 + GB34 vs. IS 50%FMWT ipsilateral side, *P* = 0.0003, 50%FMWT contralateral side, *P* < 0.0001) (Figures 3A,B), extracephalic mechanical (GB20 + GB34 vs. IS 50%PMWT ipsilateral side, *P* = 0.0009; 50%PMWT contralateral side, *P* = 0.0003) (Figures 3C,D), and extracephalic thermal hyperalgesia (GB20 + GB34 vs. IS TFL, *P* = 0.002; HPL, *P* < 0.0001; CPB, *P* = 0.0005) (Figures 3E–G), respectively. The following experiments were all performed at GB20 + GB34.

For cephalic (facial) mechanical hyperalgesia (50%FMWT), the rats of the IS group developed cutaneous mechanical hyperresponsiveness, which from days 1 to 8 on the ipsilateral side (IS vs. Con 50%FMWT, *P* < 0.0001) (Figure 1B). For extracephalic mechanical (50%PMWT) and thermal hyperalgesia (TFL, HPL, and CPB), on days 5 and 7, the pain (cutaneous hyperalgesia) thresholds for the rats’ given IS were significantly lower than those given aCSF (IS vs. Con 50%PMWT ipsilateral side, *P* < 0.0001; TFL, *P* < 0.0001; HPL, *P* < 0.0001; CPB, *P* < 0.0001) (Figures 1D,F–H). On day 8, the pain thresholds were sharply recovered to the baseline level (IS vs. Con 50%PMWT ipsilateral side, *P* = 0.1395; TFL, *P* = 0.2487; HPL, *P* = 0.3931; CPB, *P* = 0.6602) (Figures 1D,F–H). This is an interesting result of the study that we will discuss later. The above effects were also observed on the contralateral side (IS vs. Con 50%FMWT, *P* < 0.001; 50%PMWT, *P* < 0.001) (Figures 1C,E). These results indicated that the rats developed mechanical and thermal hyperalgesia after repeated IS infusion.

Electroacupuncture treatment at GB20 + GB34 have shown to reliably inhibit the IS-induced decrease in 50%FMWT on day 1 (ipsilateral side, *P* = 0.0015; contralateral side, *P* = 0.0006), day 3 (ipsilateral side, *P* < 0.0001; contralateral side, *P* = 0.0002), day 5 (ipsilateral side, *P* < 0.0001; contralateral side, *P* < 0.0001), day 7 (ipsilateral side, *P* = 0.0001; contralateral side, *P* < 0.0001), and day 8 (ipsilateral side, *P* < 0.0001; contralateral side, *P* < 0.0001) (Figures 1B,C), and blocked the IS-induced reduction in 50%PMWT on day 7 (ipsilateral side, *P* = 0.0004; contralateral side, *P* = 0.0001) (Figures 1D,E) on both the ipsilateral and contralateral sides. Furthermore, EA significantly decreased the thermal hypersensitivity on day 5 (IS + EA vs. IS TFL, *P* = 0.0002; HPL, *P* < 0.0001; CPB, *P* = 0.0189) and day 7 (IS + EA vs. IS TFL, *P* = 0.001; HPL, *P* < 0.0001; CPB, *P* = 0.0002) (Figures 1F–H). There was no significant difference between the IS + SEA group and the IS group in mechanical (50%FMWT ipsilateral side, *P* = 0.1155, contralateral side *P* = 0.8548; 50%PMWT ipsilateral side, *P* = 0.2632, contralateral side *P* = 0.1286) (Figures 1B–E) and thermal hyperalgesia (TFL, *P* = 0.3858; HPL, *P* = 0.0859; CPB, *P* = 0.9555) (Figures 1F–H). These results indicated that EA could relieve the IS-induced mechanical and thermal hyperalgesia.

The fluorescence intensity of CGRP in the Con and IS group was also tested (see Supplementary Figure 4A–D). Compared with Con group, the expression of CGRP in IS increased by about 31% and 22.5% in TG (*P* = 0.0008) and TNC (*P* = 0.0008), respectively. Based on the changes in the pain threshold and the detection of CGRP in IS rats, the construction of our recurrent migraine model was successful. Furthermore, EA treatment significantly reduced the CGRP immunofluorescence expression compared with the IS group (TG, *P* = 0.0007; TNC, *P* = 0.0032).

### Electroacupuncture Prevented Neuronal Sensitization in TNC

As previously studied, repeated IS-induced persistent sensitization also manifests as enduring enlargement of receptive fields, increases in spontaneous activity, enhancement of Von Frey filament-evoked responses and reduction in mechanical thresholds (Boyer et al., 2014). Using electrophysiological recordings, we tested whether EA treatment can prevent such persistent neuronal changes.

Twenty-four trigeminovascular WDR neurons (one neuron/animal) receiving convergent input from the dura and facial (cephalic) skin, within the TNC of Con (*n* = 6), IS rats (i.e., that had already received four IS injections, that is at 2 h after 4th IS injections; *n* = 6), sham EA- (*n* = 6) and EA-treated rats (*n* = 6) were recorded. When tested before anesthesia, sham EA treated, but not EA treated, rats exhibited a cephalic (facial region) and extracephalic (hind paw) mechanical and thermal hyperalgesia. Recorded neurons were all located within the deep laminae (IV–VI) of TNC (Figure 2B). When percutaneous electrical stimuli were applied to the center of the cutaneous receptive field of these WDR neurons, responses attributable to peripheral activation of C fibers could be observed for all recorded neurons. Mean latencies of C-fiber-evoked responses were 52.33 ± 6.302 ms in Con rats and 49.67 ± 5.76 ms in IS rats (Figure 2D). Computed conduction velocities (∼0.4–0.5 m s^–1^) were in the range of those previously reported for C fibers (Raboisson et al., 1995; Burstein et al., 1998).

Neurons in rats from the Con group exhibited little or no spontaneous activity (1.35 ± 0.2299 spikes s^–1^, Figures 2C,E). Low-intensity electrostimulation (LES)-evoked responses, 5.833 ± 0.8333 spikes s^–1^ (Figures 2F,H), and high-intensity electrostimulation (HES)-evoked responses, 9.167 ± 1.537 spikes s^–1^ (Figures 2G,H). Compared with neurons in Con rats, neurons in 4th IS rats presented stronger spontaneous activity (9.267 ± 0.5204 spikes s^–1^, *P* = 0.003948; Figures 2C,E), LES-evoked responses (16.67 ± 2.108 spikes s^–1^, *P* = 0.003353; Figures 2F,I), and HES-evoked responses (26.67 ± 2.108 spikes s^–1^, *P* = 0.003403; Figures 2G,I). These results indicate that trigeminovascular WDR neurons become sensitized following repeated activation of dural nociceptors.

In sham EA-treated rats, neurons exhibited high spontaneous activity (8.542 ± 0.5463 spikes s^–1^; Figures 2C,E) and large LES- and HES-evoked responses (15.83 ± 2.386 and 25 ± 2.582 spikes s^–1^, respectively; Figures 2F,G,J). This suggests that the responses of WDR neurons were not changed after repeated sham EA. On the other hand, compared with sham EA-treated rats, WDR neurons recorded in EA-treated rats exhibited lower spontaneous activities (3.008 ± 0.3957 spikes s^–1^, *P* = 0.003948; Figures 2C,E), smaller LES- (9.167 ± 1.537 spikes s^–1^, *P* = 0.044277; Figures 2F,K), and HES-evoked responses (15.83 ± 2.386 spikes s^–1^, *P* = 0.033118; Figures 2G,K). Thus, EA appears to completely prevent the sensitization of WDR neurons induced by repeated IS injections. Together, these data demonstrate that EA acquired antihyperalgesic properties by decreasing TNC nociceptive activity in response to IS inflammation.

### Electroacupuncture Significantly Reduced the Expression of 5-HT_7_R in TG and TNC

**FIGURE 4:**
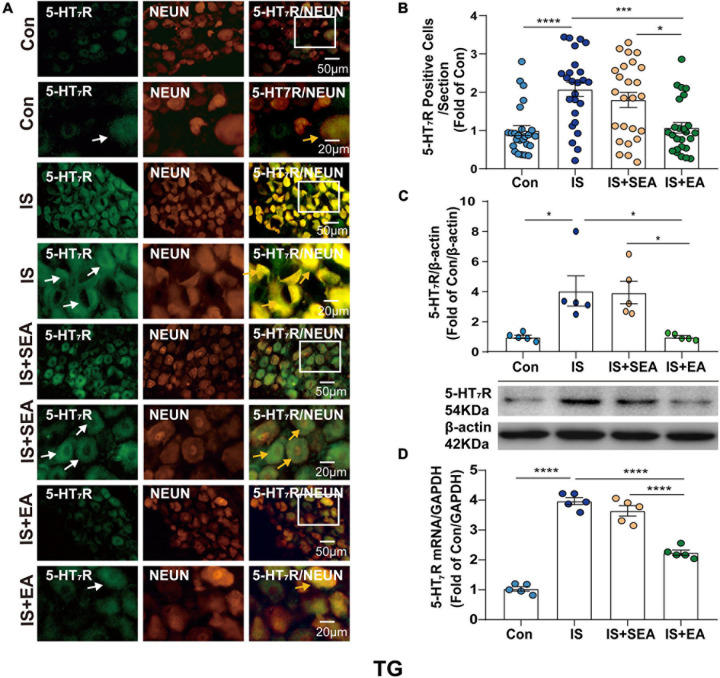
Effects of EA on the endogenous 5-HT_7_R in the TG on day 8 after the 4th IS injection. **(A)** Immunofluorescence showed that 5-HT_7_R (green) was expressed mostly in the individual neurons (NeuN, Neuron marker, red) of the TG. The upper panel displays the expression of 5-HT_7_R at low magnification, and the lower panel displays the expression of 5-HT_7_R in the TG at high magnification in each group. The white and orange arrows point to the positive cells in TG. Scale bars = 50 or 20 μm. **(B)** Quantitative analyses of 5-HT_7_R to evaluate the numbers of positive cells (*n* = 5, five sections/animal). **(C)** Representative western blot bands and quantitative analyses of 5-HT_7_R. The same membranes were probed for 5-HT_7_R with β-actin; (*n* = 5). **(D)** The mRNA levels of 5-HT_7_R were also accessed by real-time polymerase chain reaction, and values were corrected by GAPDH in TG (*n* = 5). One-way ANOVA followed by *post hoc* Tukey test. Group values are indicated by mean ± SEM. *P < 0.05, ***P < 0.001, ****P < 0.0001. 5-HT_7_R, 5-hydroxytryptamine (5-HT)_7_ receptor; GAPDH, glycolytic enzyme glyceraldehyde 3-phosphate dehydrogenase; TG, trigeminal ganglion; EA, electroacupuncture; IS, inflammatory soup; SEA, sham electroacupuncture; Con, control.

**FIGURE 5:**
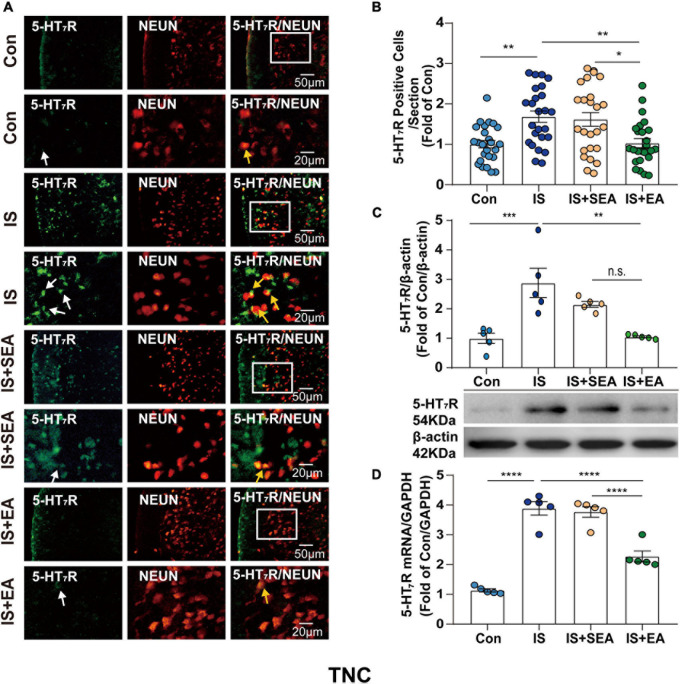
Effects of EA on the endogenous 5-HT_7_R in the TNC on day 8 after the 4th IS injection. **(A)** Immunofluorescence showed that 5-HT_7_R (green) was expressed mostly in the individual neurons (NeuN, neuron marker, red) of the TNC. The upper panel displays the expression of 5-HT_7_R at low magnification, and the lower panel displays the expression of 5-HT_7_R in the TNC at high magnification in each group. The white and orange arrows point to the positive cells in TNC. Scale bars = 50 or 20 μm. **(B)** Quantitative analyses of 5-HT_7_R to evaluate the numbers of positive cells (*n* = 5, five sections/animal). **(C)** Representative western blot bands and quantitative analyses of 5-HT_7_R. The same membranes were probed for 5-HT_7_R with β-actin; (*n* = 5). **(D)** The mRNA levels of 5-HT_7_R were also accessed by real-time polymerase chain reaction, and values were corrected by GAPDH in TNC (*n* = 5). One-way ANOVA followed by *post hoc* Tukey test. Group values are indicated by mean ± SEM. *P < 0.05, **P < 0.01, ***P < 0.001, ****P < 0.0001. 5-HT_7_R, 5-hydroxytryptamine (5-HT)_7_ receptor; TNC, trigeminal nucleus caudalis; EA, electroacupuncture; IS, inflammatory soup; SEA, sham electroacupuncture; Con, control.

To determine whether the effects of EA were associated with 5-HT_7_R, we performed RT-PCR, Western blot, and immunofluorescence analyses. Samples from TG and TNC were respectively collected on day 8 after the 4th IS injection. There were significant changes in the 5-HT_7_R mRNA expression in response to IS injection compared with the Con group in TG and TNC (TG, *P* < 0.0001; TNC, *P* < 0.0001). However, the 5-HT_7_R mRNA level of the IS + EA group was significantly reduced compared with that of the IS group in TG and TNC (TG, *P* < 0.0001; TNC, *P* < 0.0001) (Figures 4D, 5D). Next, we examined the total protein content of 5-HT_7_R in Con, IS, IS + SEA, and IS + EA groups by Western blot analysis. Significant differences were observed in both TG and TNC between the IS group and the Con group (TG, *P* = 0.0165; TNC, *P* = 0.0007). EA treatment significantly reduced the 5-HT_7_R protein content compared with the IS group (TG, *P* = 0.0163; TNC, *P* = 0.0009) (Figures 4C, 5C). We also used immunofluorescence double labeling to detect the distribution of 5-HT_7_R in TG and TNC and found that 5-HT_7_R were colocalized with neurons (Figures 4A, 5A, 3rd column, orange arrowheads). IF analysis revealed increased 5-HT_7_R-positive cells were double positive for NeuN in IS group compared with the Con group, and 5-HT_7_R proteins were expressed in large-sized TG neurons in immunostaining of TG sections (Figure 4A, 1st column, white arrowheads). Consistent with the Western blot results, the 5-HT_7_R level seemed to reduce in the IS + EA group compared with the IS group (TG, *P* = 0.0003; TNC, *P* = 0.0037) (Figures 4A,B, 5A,B). There were significant differences between the IS + EA and IS + SEA group in the abovementioned test (RT-PCR: TG, *P* < 0.0001; TNC, *P* < 0.0001; WB: TG, *P* = 0.0207; TNC, *P* = 0.0457; IF: TG, *P* = 0.0132; TNC, *P* = 0.0114) (Figures 4A–D, 5A–D). These results indicated that EA relieved IS-induced hyperalgesia, which is shown by a reduction in 5-HT_7_R expression.

The effects of electroacupuncture at different acupoints were also compared, including GB20 + GB34, GB20 + SJ17, and GB34 + ST36 (see Supplementary Figures 5A–E, 6A–E), in changing the 5-HT_7_R expression of IS rats. We found that EA at GB20 + GB34, GB20 + SJ17, or GB34 + ST36 reduced the 5-HT_7_R expression. The GB20 + GB34 group showed the best effect that reduced 5-HT_7_R expression.

### Electroacupuncture Inhibited the 5-HT_7_R-Associated PKA and ERK_1__/__2_ Phosphorylation Signaling Pathway in TG and TNC

**FIGURE 6:**
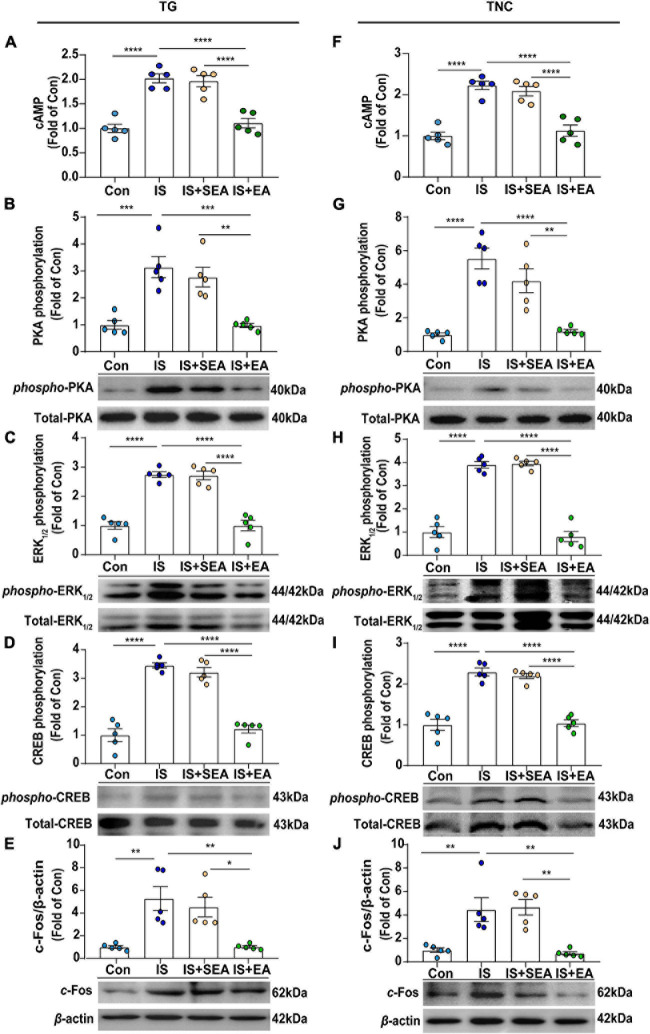
Effects of EA on the protein levels of cAMP and 5-HT_7_R-associated PKA, ERK_1__/__2_ phosphorylation signaling pathway in dural 4th IS-injected rats. Results were averages of cAMP production as evaluated by ELISA (*n* = 5) **(A,F)**. Representative Western blots of phosphorylated and total PKA **(B,G)**, extracellular signal-regulated protein kinase_1__/__2_ (ERK_1__/__2_) **(C,H)**, CREB **(D,I)**, as well as c-Fos **(E,J)**. The densitometric analysis of phosphorylated and c-Fos protein levels detected from the TG and TNC of Con, IS, IS + SEA, IS + EA. One-way ANOVA followed by *post hoc* Tukey test. Group values are indicated by mean ± SEM. *P < 0.05, **P < 0.01, ***P < 0.001, ****P < 0.0001. 5-HT_7_R, 5-hydroxytryptamine (5-HT)_7_ receptor; cAMP, cyclic adenosine monophosphate; PKA, protein kinase A; ERK_1__/__2_, extracellular signal-regulated kinase_1__/__2_; CREB, cAMP-responsive element-binding protein. TNC, trigeminal nucleus caudalis; TG, trigeminal ganglion; EA, electroacupuncture; IS, inflammatory soup; SEA, sham electroacupuncture; Con, control.

It is known that 5-HT_7_R could influence PKA- or ERK_1__/__2_-mediated signaling pathways through interacting with Gαs-cAMP (Ohta et al., 2006; Cho et al., 2015), and the increasing PKA-p and p-ERK_1__/__2_ could be blocked by EA treatment. As a result, 5-HT_7_R-related protein content of cAMP, PKA, p-PKA, ERK_1__/__2_, p-ERK_1__/__2_, CREB, and p-CREB were also detected. As we first measured the cAMP levels, a significant difference was shown between EA and IS groups (TG, *P* < 0.0001; TNC, *P* < 0.0001) (Figures 6A,F), suggesting that EA-induced 5-HT_7_R inhibition is cAMP dependent.

Next, we found that the protein expression of p-PKA (TG, *P* = 0.0003; TNC, *P* < 0.0001), p-ERK_1__/__2_ (TG, *P* < 0.0001; TNC, *P* < 0.0001), and p-CREB (TG, *P* < 0.0001; TNC, *P* < 0.0001) in IS group was increased compared with the Con group, and EA treatment inhibited p-PKA (TG, *P* = 0.0003; TNC, *P* < 0.0001), p-ERK_1__/__2_ (TG, *P* < 0.0001; TNC, *P* < 0.0001), and p-CREB (TG, *P* < 0.0001; TNC, *P* < 0.0001) expression (Figures 6B–D,G–I). However, there was no significant difference in total amount of PKA, ERK_1__/__2_, and CREB between the four groups (Figures 6B–D,G–I).

C-Fos, which is a classical marker of neuronal activation with trigeminovascular nociceptive pathways, could be initiated or potentiated by CREB phosphorylation (Mitsikostas et al., 2011). Here, we found that the expression of c-Fos in the IS group was increased (TG, *P* = 0.0022; TNC, *P* = 0.0056) compared with the Con group. However, the expression of c-Fos was suppressed after EA treatment (TG, *P* = 0.0022; TNC, *P* = 0.003) (Figures 6E,J). Sham EA had no effect on the expression of cAMP, p-PKA, p-ERK_1__/__2_, p-CREB, and c-Fos (IS + SEA vs. IS) (Figures 6A–J). The above results demonstrated that EA inhibited the 5-HT_7_R-associated PKA and ERK_1__/__2_ phosphorylation signaling pathways in TG and TNC.

### Antihyperalgesic Effects and Inhibition of Neuronal Sensitization Produced by Electroacupuncture Were Weakened in 5-HT_7_R Agonist AS19-Treated IS Rats and Mimicked in 5-HT_7_R Antagonist SB269970-Treated IS Rats

**FIGURE 7:**
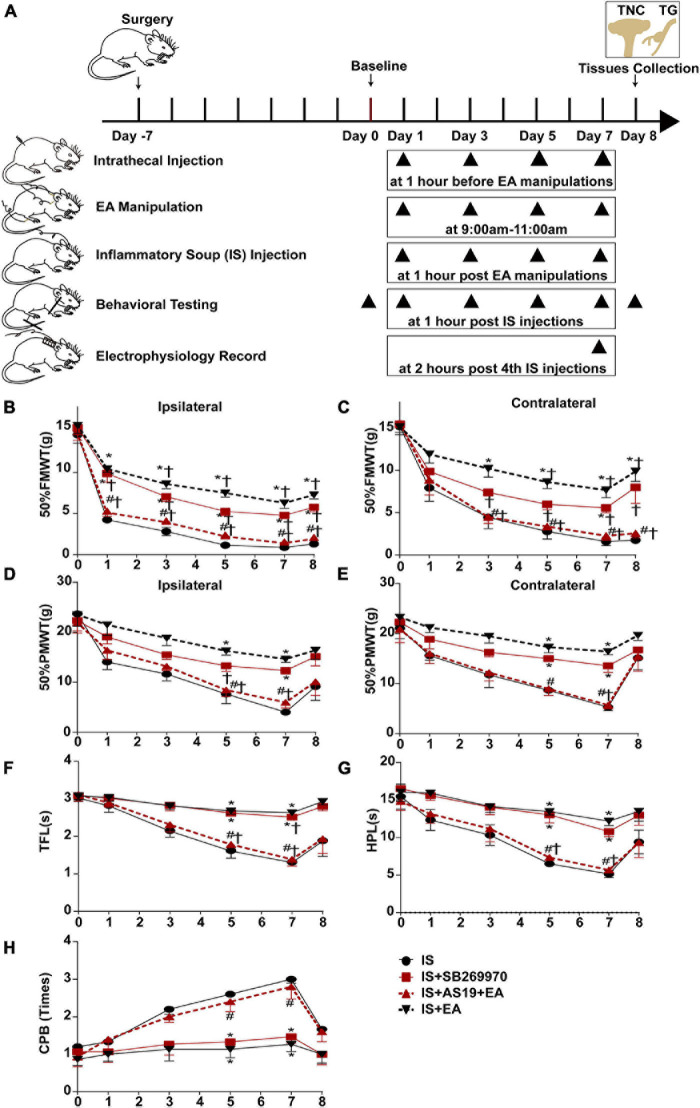
Injecting 5-HT_7_R agonist AS19 or 5-HT_7_R antagonist SB269970 into the intrathecal space of IS rats, evaluated the roles of 5-HT_7_R in EA’s antihyperalgesic effects. **(A)** Scheme of the experimental design. Rats were injected 5-HT_7_R agonist AS19 or 5-HT_7_R antagonist SB269970 into the intrathecal space, following EA manipulation application and repeated-dural IS injection on days 1, 3, 5, and 7, then measurements of 50%FMWT, 50%PMWT, TFL, HPL, and CPB to examine the extent of mechanical and thermal hyperalgesia after 1 h of IS injection, and also the day 0 without IS injection and 1 day after the 4th IS injection (day 8). Changes in mechanical [50% FMWT **(B,C)** and 50% PMWT **(D,E)**] and thermal [TFL **(F)**, HPL **(G)**, and CPB **(H)**] hyperalgesia in IS, IS + SB269970, IS + AS19 + EA, and IS + EA groups on days 0, 1, 3, 5, 7, and 8 (*n* = 5). The repeated-measure two-way ANOVA *post hoc* Tukey multiple comparisons test was used. Group values are indicated by mean ± SEM. *P < 0.05 compared with the IS group; ^#^*P* < 0.05 compared with the IS + EA group at the same time point; ^†^*P* < 0.05 compared with the baseline on day 0; 5-HT_7_R, 5-hydroxytryptamine (5-HT)_7_ receptor; 50%FMWT, 50% facial mechanical withdrawal threshold; 50%PMWT, 50% paw mechanical withdrawal threshold; TFL, tail-flick latency; HPL, hot-plate latency; CPB, cold-plate behaviors; EA, electroacupuncture; IS, inflammatory soup; AS19, 5-HT_7_R agonist; SB269970, 5-HT_7_R antagonist.

Based on our results that EA reduced the content of 5-HT_7_R, we used a pharmacological strategy to reveal the role of 5-HT_7_R in the antihyperalgesic effects and inhibition of neuronal sensitization of EA (Figure 7A). To measure the changes in cutaneous hyperalgesia and nociceptive neuron activity, the rats from EA and non-EA groups were injected with either 5-HT_7_R antagonist (SB269970) or 5-HT_7_R agonist (AS19) before being injected with IS.

**FIGURE 8:**
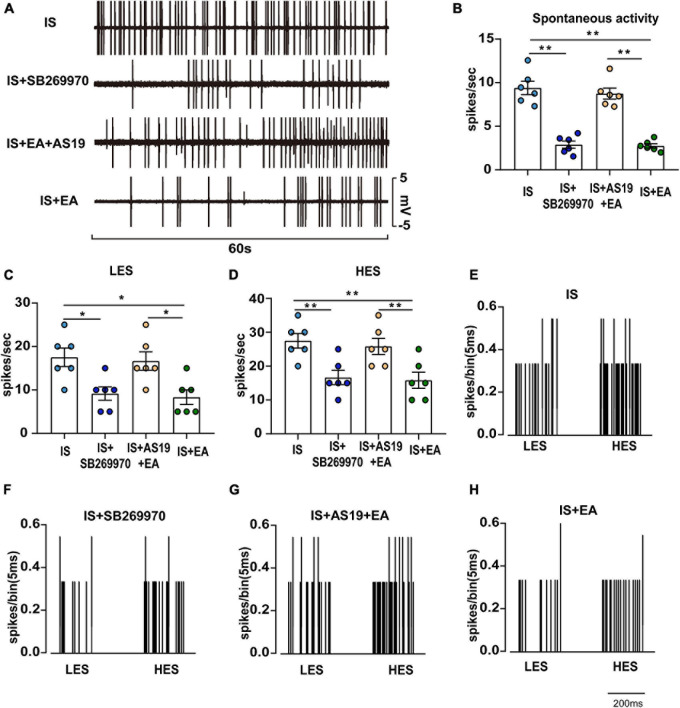
Overview of the electrophysiological recordings and neuronal characteristics among the IS, IS + SB269970, IS + AS19 + EA, and IS + EA groups. **(A)** Spontaneous activity was recorded for 60 s at 30 min postfinding the activated neurons for stabilization among the indicated groups. Spontaneous activity **(B)**, LES-evoked responses **(C)**, and HES-evoked responses **(D)** of WDR neurons recorded 2 h after the 4th IS in the TCC of none manipulation (IS and IS + SB269970) **(E,F)** and EA (IS + AS19 + EA and IS + EA) **(G,H)** rats. When four groups were compared for electrophysiology, a Kruskal-Wallis test was used, and when only two groups were compared, a Mann-Whitney *U* test was used. Group values are indicated by mean ± SEM. *P < 0.05, **P < 0.01. Tc, the stimulation intensities required to evoke neuronal activity with a conductive velocity of 0.4–2 m s^–1^, namely about 2 mA. 5-HT_7_R, 5-hydroxytryptamine (5-HT)_7_ receptor; LES, low-intensity electrostimulation; HES, high-intensity electrostimulation; WDR, wide-dynamic range; TNC, trigeminal nucleus caudalis; aCSF, artificial cerebrospinal fluid; EA, electroacupuncture; IS, inflammatory soup; AS19, 5-HT7R agonist; SB269970, 5-HT7R antagonist.

Injection of IS induced a significant decrease of hyperalgesia intensity in SB269970-treated rats on day 7 (IS + SB269970 vs. IS mechanical hyperalgesia: 50%FMWT ipsilateral side, *P* = 0.0046; 50%FMWT contralateral side, *P* = 0.0210, 50%PMWT ipsilateral side, *P* = 0.0023; 50%PMWT contralateral side, *P* = 0.0165; thermal hyperalgesia: TFL, *P* = 0.0013; HPL, *P* = 0.0333; CPB, *P* = 0.0029) (Figures 7B–H), but such differences were absent in IS + SB269970 and IS + EA rats (IS + SB269970 vs. IS + EA mechanical hyperalgesia: 50%FMWT ipsilateral side, *P* = 0.1047; 50%FMWT contralateral side, *P* = 0.5108, 50%PMWT ipsilateral side, *P* = 0.2588; 50%PMWT contralateral side, *P* = 0.2287; thermal hyperalgesia: TFL, *P* = 0.193; HPL, *P* = 0.5863; CPB, *P* = 0.6699) (Figures 7B–H). Compared with IS rats (Figures 8A–E), WDR neurons recorded in IS + SB269970 rats exhibited lower spontaneous activities (2.892 ± 0.4142 spikes s^–1^, *P* = 0.003948; Figures 8A,B), smaller LES- (9.167 ± 1.537 spikes s^–1^, *P* = 0.016864; Figures 8C,F) and HES-evoked responses (16.67 ± 2.108 spikes s^–1^, *P* = 0.0065; Figures 8D,F). The electrophysiological properties of IS + SB269970 rats were similar to those of IS + EA rats (spontaneous activities, *P* = 0.8728; LES-evoked responses; *P* = 0.6654; HES-evoked responses *P* = 0.7370, Figures 8A–D,H). Together, these data indicated that SB269970 mimics the antihyperalgesic effects and inhibition of neuronal sensitization of EA.

The hyperalgesia intensity of the IS + AS19 + EA group was significantly aggravated when compared against the IS + EA group (IS + AS19 + EA vs. IS + EA mechanical hyperalgesia: 50%FMWT ipsilateral side, *P* = 0.0074; 50%FMWT contralateral side, *P* = 0.0069, 50%PMWT ipsilateral side, *P* = 0.0124; 50%PMWT contralateral side, *P* = 0.0002; thermal hyperalgesia: TFL, *P* = 0.0095; HPL, *P* < 0.0001; CPB, *P* = 0.0363), whereas no differences were seen between the IS and IS + AS19 + EA groups (Figures 7B–H). Low spontaneous activities, small LES- and HES-evoked responses in IS + EA rats were activated by AS19 (IS + AS19 + EA vs. IS + EA spontaneous activities, *P* = 0.0039; LES-evoked responses, *P* = 0.0165; HES-evoked responses, *P* = 0.0049) (Figures 8B–D,G,H). Together, these data indicated that the antihyperalgesic effects and inhibition of neuronal sensitization produced by EA treatment were weakened in AS19-treated IS rats. The abovementioned finding suggested that 5-HT_7_R is necessary for EA antihyperalgesia and inhibition of neuronal sensitization.

### 5-HT_7_R-Associated PKA and ERK_1__/__2_ Phosphorylation Through cAMP Signaling May Be Involved in EA’s Antihyperalgesic Effects

**FIGURE 9:**
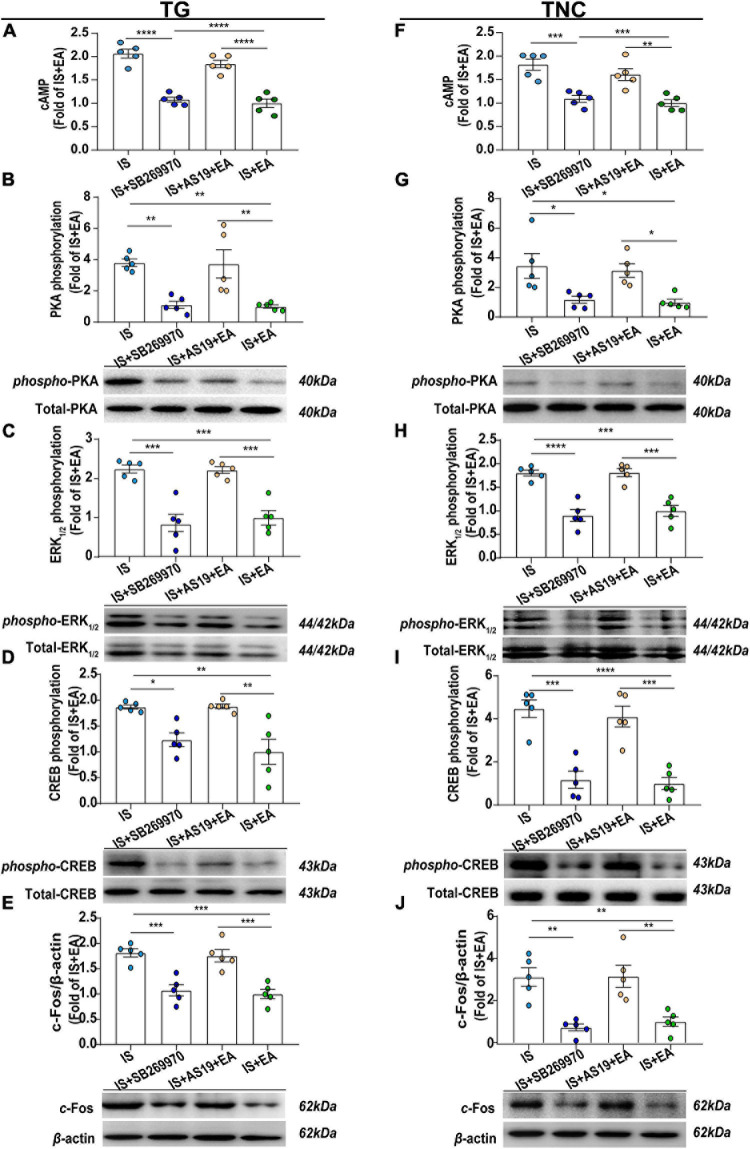
Roles of 5-HT_7_R-associated PKA, ERK_1__/__2_ phosphorylation signaling pathway in EA’s antihyperalgesic effects. Results were averages of cAMP production as evaluated by ELISA (*n* = 5) **(A,F)**. Representative Western blots of phosphorylated and total PKA **(B,G)**, ERK_1__/__2_ **(C,H)**, and CREB **(D,I)**, as well as c-Fos **(E,J)**. The densitometric analysis of phosphorylated and c-Fos protein levels detected from the TG and TNC of IS, IS + SB269970, IS + AS19 + EA, and IS + EA. One-way ANOVA followed by *post hoc* Tukey test. Group values are indicated by mean ± SEM. *P < 0.05, **P < 0.01, ***P < 0.001, ****P < 0.001. 5-HT_7_R, 5-hydroxytryptamine (5-HT)_7_ receptor; PKA, protein kinase A; ERK_1__/__2_, extracellular signal-regulated kinase_1__/__2_; CREB, cAMP-responsive element-binding protein; TG, trigeminal ganglion; TNC, trigeminal nucleus caudalis; EA, electroacupuncture; IS, inflammatory soup; AS19, 5-HT7R agonist; SB269970, 5-HT7R antagonist.

The behavioral analysis showed that the activation of 5-HT_7_R weakened but did not completely abolish the antihyperalgesic effects of EA. To explore the reason why the antihyperalgesic effects of EA were weakened in the AS19-treated IS rats, we examined the protein content of cAMP, PKA, p-PKA, ERK_1__/__2_, p-ERK_1__/__2_, CREB, p-CREB, and c-Fos, which plays a pivotal role in the 5-HT_7_R-mediated transmission of nociceptive information. Electroacupuncture reduced the cAMP, p-PKA, p-ERK_1__/__2_, p-CREB, and c-Fos levels to the baseline level in IS rats (IS vs. IS + EA) (Figures 9A–J). Electroacupuncture significantly downregulated cAMP, p-PKA, p-ERK_1__/__2_, p-CREB, and c-Fos expression in IS rats but not in AS19-treated IS rats (IS + EA vs. IS + AS19 + EA, cAMP: TG, *P* < 0.0001; TNC, *P* = 0.0029; p-PKA: TG, *P* = 0.0053; TNC, *P* = 0.0364; p-ERK_1__/__2_: TG, *P* = 0.0006; TNC, *P* = 0.0002; p-CREB: TG, *P* = 0.0024; TNC, *P* = 0.0003;c-Fos: TG, *P* = 0.0005; TNC, *P* = 0.0039) (Figures 9A–J). The results suggested that 5-HT_7_R activation likely weakened the regulation of cAMP, p-PKA, p-ERK_1__/__2_, p-CREB, and c-Fos by EA but did not completely inhibit the effect. Also, EA did not affect the content of PKA, ERK_1__/__2_, and CREB in both IS and AS19-treated IS rats (Figures 9B–D,G–I). These results indicate that AS19 impaired the suppression effects of EA on endogenous cAMP, p-PKA, p-ERK_1__/__2_, p-CREB, and c-Fos, which could explain that the antihyperalgesic effects of EA were weakened in the AS19-treated IS rats.

Then, we detected the effects of the 5-HT_7_R antagonist (SB269970) on the above protein levels. It could inhibit cAMP, p-PKA, p-ERK_1__/__2_, and p-CREB expression in TG and TNC of IS rats by injecting 7 μl of SB269970 (5 mM) into the intrathecal space (cAMP: TG, *P* < 0.0001; TNC, *P* = 0.0005; p-PKA: TG, *P* = 0.0059; TNC, *P* = 0.0256; p-ERK_1__/__2_: TG, *P* = 0.0001; TNC, *P* < 0.0001; p-CREB: TG, *P* = 0.0282; TNC, *P* = 0.0001) (Figures 9A–D,F–I). This is consistent with the results that EA decreases the expression of 5-HT_7_R-mediated signaling pathways related protein. Then, SB269970 inhibited p-CREB-mediated transcriptional regulation factor c-Fos (TG, *P* = 0.0006; TNC, *P* = 0.0016) (Figures 9E,J). In all, 5-HT_7_R antagonist mimics the antihyperalgesic effects of EA and inhibited p-PKA, p-ERK_1__/__2_, and p-CREB. There was also no statistical difference in PKA, ERK_1__/__2_, and CREB (Figures 9B–D,G–I). Based on the results, we concluded that 5-HT_7_R-associated PKA and ERK_1__/__2_ phosphorylation signaling pathways may be involved in EA antihyperalgesia.

## Discussion

Our study demonstrates that EA decreased IS-induced elevated content of 5-HT_7_R in the TG and TNC, suggesting that there is a certain relationship between 5-HT_7_R expression and EA treatment. 5-HT_7_R agonist AS19 impaired the antihyperalgesic effects of EA on p-PKA and p-ERK_1__/__2_. Injecting 5-HT_7_R antagonist SB-269970 into the intrathecal space of IS rats mimicked the effects of EA antihyperalgesia and inhibited p-PKA and p-ERK_1__/__2_, indicating decreased 5-HT_7_R expression and decreased p-PKA and p-ERK_1__/__2_ expression are sufficient to EA treatment.

**FIGURE 10:**
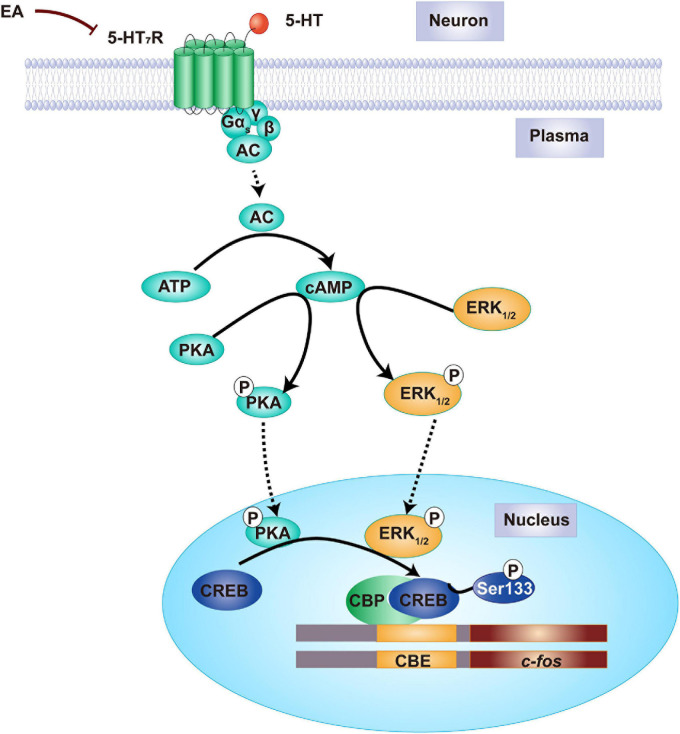
Proposed mechanism of the antihyperalgesic effects of EA stimulation. Before EA, there is a relatively high amount of 5-HT_7_R protein in TG and TNC of IS rats. The medial cAMP is formulated in the cytoplasm of neuron, resulting in PKA and ERK_1__/__2_ phosphorylation. Once PKA and ERK_1__/__2_ are phosphorylated into p-PKA and p-ERK_1__/__2_, the CREB will move from the cytoplasm into the nuclear and phosphorylated at Ser133. Following nuclear CREB-CBP elevation, the initiate or potentiate of early gene *c-fos* was regulated by p-CREB-mediated transcription, resulting in dural IS stimulation that leads to hyperalgesia. By contrast, EA treatment decreases the amount of 5-HT_7_R proteins, forming lower cAMP, thereby PKA and ERK_1__/__2_ phosphorylation is inhibited in the cytoplasm. This prevents CREB phosphorylation, and therefore gene *c-fos* silence, ultimately producing an antihyperalgesic effect. 5-HT_7_R, 5-hydroxytryptamine (5-HT)_7_ receptor; cAMP, cyclic adenosine monophosphate; PKA, protein kinase A; ERK_1__/__2_, extracellular signal-regulated kinase_1__/__2_; CREB, cAMP-responsive element-binding protein; EA, electroacupuncture.

As it is known that 5-HT_7_R-associated PKA and ERK_1__/__2_ activation is dependent on Gαs-cAMP signaling in the nervous system, we speculated the potential mechanisms underlying EA as follows (Figure 10). Upon IS dural injection, 5-HT_7_R is stimulated to couple positively with adenylate cyclase (AC) through activating Gαs, resulting in an increase of cAMP to ultimately phosphorylate PKA and ERK_1__/__2_. Phosphorylation of PKA and ERK_1__/__2_ causes the nuclear translocation of CREB so that making it activation by phosphorylation at Ser133. The consequent increase in nuclear CREB-CBP complex in TG and TNC might initiate or potentiate p-CREB-mediated transcriptional regulation of immediate early gene *c-fos* that is sufficient to neuronal activation with trigeminovascular nociceptive pathways (Mitsikostas et al., 2011). Upon EA treatment, the 5-HT_7_R function is decreased, thus leading to a decrease in the content of cAMP which results in a reduction in the formation of p-PKA and p-ERK_1__/__2_ and their respective retention in the cytoplasm, which then attenuates induced CREB phosphorylation and transcriptional pronociceptive gene (Figure 10). Ultimately, a series of the abovementioned processes would not occur under EA treatment. Then, the trigeminovascular nociceptive sensitization could be unsustainable. Therefore, our results suggest that 5-HT_7_R mediates the antihyperalgesic effects of EA and indicates that the 5-HT_7_R-associated PKA and ERK_1__/__2_ phosphorylation signaling may be a new underlying mechanism.

However, how EA regulates 5-HT_7_R remains unclear. Electroacupuncture was reported to reduce the cytoplasmic content of cAMP (Shao et al., 2016). Decreased cAMP affects the phosphorylation of PKA and ERK_1__/__2_ (Skalhegg and Tasken, 2000; Zanassi et al., 2001). Given the interaction between Gαs-cAMP and 5-HT_7_R, the possibility of EA affecting 5-HT_7_R exists. This specific mechanism would be a direction for our further research. Notably, the antihyperalgesic effects of EA were weakened but not completely obliterated in the activation of 5-HT_7_R, due to the presence of the other potential mechanisms. Given that EA could influence multiple signaling pathways, including β-endorphins (Han, 2003), cannabinoid CB2 receptors, and adenosine A1 receptor (Goldman et al., 2010), the 5-HT_7_R-associated PKA and ERK_1__/__2_ phosphorylation through Gαs-cAMP signaling is likely only one of the key factors involved in EA’s maintenance of antihyperalgesic effects. Moreover, cAMP interacts with several other proteins in addition to PKA and ERK_1__/__2_, including ROS (Gu et al., 2018), NF-κB (Wang J. et al., 2018), and AKAP (Miyano et al., 2019), which might also be involved in the maintenance of the antihyperalgesic effects by EA. Next, we will use naloxone to block opioid receptors, or DPCPX to block A1 receptors, so as to exclude the effect of other factors on EA antihyperalgesia, and then confirm the role of 5-HT_7_R.

We found that the extracephalic pain thresholds of IS group were sharply recovered to the baseline level on day 8, but there were significantly lower in the IS group compared with the Con group on days 5 and 7 (Figures 1D–H). However, the IS group maintained persistent cephalic static mechanical hyperalgesia from days 1 to 8 (Figures 1B,C). These results were consistent with the previous investigation on the effect of IS on cephalic and extracephalic cutaneous hyperalgesia. Boyer et al. (2014) found that repeated IS injection (four times IS protocol) developed reversible extracephalic and persistent cephalic hyperalgesia. Specifically, extracephalic pain thresholds decreased 1 h after the 4th IS injection and returned to pre-IS values within 2–3 h after the 4th IS injection. Cephalic pain thresholds before the 4th IS injection were already lower than those in the Con group. Similarly, we found rats developed lower extracephalic pain thresholds after 1 h of 3rd and 4th IS injections on days 5 and 7 and returned to the baseline level on day 8 without IS injection. Furthermore, the repeats of IS produced ever stronger and longer cephalic hyperalgesia from days 1 to 8. Such repetition of IS-induced development of central sensitization and its consequence, cutaneous hyperalgesia, may arise from hyperexcitability that likely develops in trigeminal nociceptive neurons in response to their repetitive activation (Figures 2C,E). The sensitization of trigeminal meningeal nociceptors in migraine is strongly related to meningeal neurogenic inflammation with a cascade of events such as neuropeptides release (e.g., CGRP and SP) (Hanci et al., 2021). CGRP is a potent vasodilator leading to vasodilation in the dura mater during a migraine attack (Hanko et al., 1985; Kilinc et al., 2018), while substance P shows a far less potent vasoactive effect compared with CGRP, but it is the prime mediator that regulates plasma protein extravasation secondary to capillary leakage (Markowitz et al., 1988). These two substances can both induce dural mast cell degranulation (Ottosson and Edvinsson, 1997), which further aggravates the neurogenic inflammation through releasing multiple proinflammatory and pronociceptive molecules to residents such as CGRP, substance P, 5-HT, prostaglandins, bradykinin, histamines, and cytokines, eventually leading to the initiation of migraine (Koyuncu Irmak et al., 2019; Theoharides et al., 2019). EA exerted antihyperalgesic effects, at least in part, *via* preventing the maintenance of a state of facilitated trigeminovascular transmission within the TNC. Both Aδ fibers and C fibers contribute to migraine-related nociception in the origin site of the migraine pain (Zakharov et al., 2015; Kilinc et al., 2017); however, compared with the Aδ fibers, the activation of C-afferent fibers by electroacupuncture triggers stronger analgesic effects (Kawakita and Funakoshi, 1982; Xu et al., 2003; Zhu et al., 2004). Based on gate control theory (Melzack and Wall, 1965), the substantia gelatinosa between primary afferents and projection neurons in the dorsal horn functions as a gate control system that turns on or off the gate. The activation of Aδ fibers fires the cells of the substantia gelatinosa (SG cells), reducing the activities of the projection neurons (T cells) by presynaptic inhibition, then closing the gate, which decreases the transmission of pain sensation toward the brain. However, the activation of C fibers inhibits SG cells, making the gate open, which allows the transmission of pain sensation toward the brain, worsening the situation. The interaction between large-diameter fibers and small-diameter fibers determines the opening and closing of the gate. The gate control theory has modified with the development of molecular biology and the researches of chronic pain, emphasizing the diversity of SG cells (excitability and inhibition), and SG cells have both presynaptic and postsynaptic inhibition for T cells.

The incessant discharge of T cells, also called the wind-up effect, will be induced when extremely intense pain is caused by C fibers or C fibers are activated repeatedly, which results from the constant inhibition of SG cells, known as the pain signal caused by C fiber will not produce “adaptation” phenomenon. Meanwhile, the analgesic effects of Aδ fibers disappear, and the central descending activities occupy the key position in regulating the gate. T cells are able to trigger the central control system to transmit the impulse when the impulse exceeds a certain threshold, regulating the gate by presynaptic inhibition (McMahon and Wall, 1983). The activation of C fibers by electroacupuncture may promote the T cells’ impulse to exceed a certain threshold.

Based on a previous experience (Ye et al., 2017; Cui et al., 2019; Hu et al., 2020), we performed sham EA manipulation to ensure the 5-HT_7_R is specific in EA antihyperalgesia. The results showed that 5-HT_7_R expression was not decreased in the IS + SEA group (Figures 4A–D, 5A–D), cAMP was not decreased (Figures 6A,F), and there was no statistical difference in p-PKA, p-ERK_1__/__2_, p-CREB, and c-Fos compared with the IS group (Figures 6B–E,G–J), indicating that sham EA had no effect on 5-HT_7_R and its related protein, and that EA was specific.

Previous studies have demonstrated the existence of two different mechanisms for pro- or antinociceptive actions of 5-HT. The robust pronociceptive firing together with CGRP release in the peripheral nerve terminals and the antinociceptive presynaptic inhibition of the central nerve terminal were induced by 5-HT through the same 5-HT_3_R in various migraine models (Kilinc et al., 2017). Like 5-HT_3_R activation, 5-HT_7_R can also present pro- or antinociceptive actions according to receptor location and/or quality and modality of the stimulus in neuropathic pain models (Hedlund et al., 2010; Yanarates et al., 2010; Viguier et al., 2012). In our study, we found that activation of 5-HT_7_R may play a pronociceptive role in both central (TNC) and peripheral (TG). The discrepancy may be due to the different locations of the serotonin receptor in the pain transmission pathway and the different pain-related animal models that were researched. Besides, studies (Ohta et al., 2006; Cho et al., 2015) show that the PKA- or ERK_1__/__2_-mediated signaling pathways are involved in the complete Freund’s adjuvant or capsaicin-induced long-lasting pain hypersensitivity through 5-HT_7_R. More expression of p-PKA or p-ERK_1__/__2_ means more excitation of dura-sensitive trigeminal neurons and more sensitization of pain-transducing receptors (Ji, 2004; Giniatullin et al., 2008). Indeed, there were significant differences in the expression of p-PKA and p-ERK_1__/__2_ between the IS + SB269970 group and the IS group, which is consistent with the previous study. We found that 5-HT_7_R agonist AS19 could recover EA-induced decreased cAMP and inhibited p-PKA and p-ERK_1__/__2_ in the TG and TNC. 5-HT_7_R antagonist SB-269970 generated the antihyperalgesic effects on neurogenic inflammation pain induced by IS, which mimics the effects of EA antihyperalgesia. In addition, we found that the expression of cAMP protein increased with the activation of 5-HT_7_R, which confirmed the interaction between Gαs-cAMP and 5-HT_7_R. It indicated 5-HT_7_R as a new target for enhancing the EA antihyperalgesic efficacy in migraine, and the development and application of related drugs could be promoted furthermore.

Based on our findings, the antihyperalgesic effects of EA have been further verified. As it is now established that 5-HT_7_R affects the phosphorylation of PKA and ERK_1__/__2_ through Gαs-cAMP, this interaction could be involved in the facilitation of persistent pain and regulation of peripheral/central sensitization, further highlighting the theory that 5-HT_7_R mediates the antihyperalgesic effects of EA. Further explorations of the functions and roles of 5-HT_7_R may reveal new insight into the mechanisms underlying migraine.

In summary, our results indicate that 5-HT_7_R mediates the antihyperalgesic effects of EA on IS-induced neurogenic inflammation pain by regulating PKA and ERK_1__/__2_ phosphorylation through cAMP. Our study may provide a new target to enhance the EA antihyperalgesic effects in migraine.

## Data Availability Statement

The original contributions presented in the study are included in the article/Supplementary Material, further inquiries can be directed to the corresponding author/s.

## Ethics Statement

The animal study was reviewed and approved by Animal Experimentation Ethics Committee of Beijing Institute of Traditional Chinese Medicine.

## Author Contributions

LL, X-HJ, and BL designed the research study. LL, X-BX, Z-YQ, L-PZ, Z-JL, T-LL, and X-FW performed the experiment. C-SZ analyzed the data. LL analyzed the results and wrote this manuscript. All authors read and approved the final manuscript.

## Conflict of Interest

The authors declare that the research was conducted in the absence of any commercial or financial relationships that could be construed as a potential conflict of interest. The handling editor declared a past co-authorship with one of the authors, X-HJ.
