# Supplementary material for: Determining 5HT7R’s Involvement in Modifying the Antihyperalgesic Effects of Electroacupuncture on Rats With Recurrent Migraine
Source: Front Neurosci. 2021 Jun 7;15:668616. doi: 10.3389/fnins.2021.668616 (PMC8215279; doi:10.3389/fnins.2021.668616)
Supplement: Supplementary file 1 [file Data_Sheet_1.pdf]

Supplementary material

Supplementary Table 1. Acupoints and EA manipulations

Acupoints	Anatomical positions	EA manipulation	Times (min)
Fengchi (GB20)	3 mm from the midpoint of the two ears connecting lines behind the head	2-15 Hz alternating wave, 1.0 mA	15
Yanglingquan (GB34)	at the depression anterior and inferior to the head of the fibula	2-15 Hz alternating wave, 1.0 mA	15
Yifeng (SJ17)	behind the earlobe, the depression between the mastoid process and the mandible	2-15 Hz alternating wave, 1.0 mA	15
Zusanli (ST36)	at 2 mm lateral to the anterior tubercle of the tibia, and 5 mm below the capitulum fibulae under the knee joint	2-15 Hz alternating wave, 1.0 mA	15
Non-acupoints	approximately 10 mm and 15mm above the iliac crest	without electrical stimulation	15

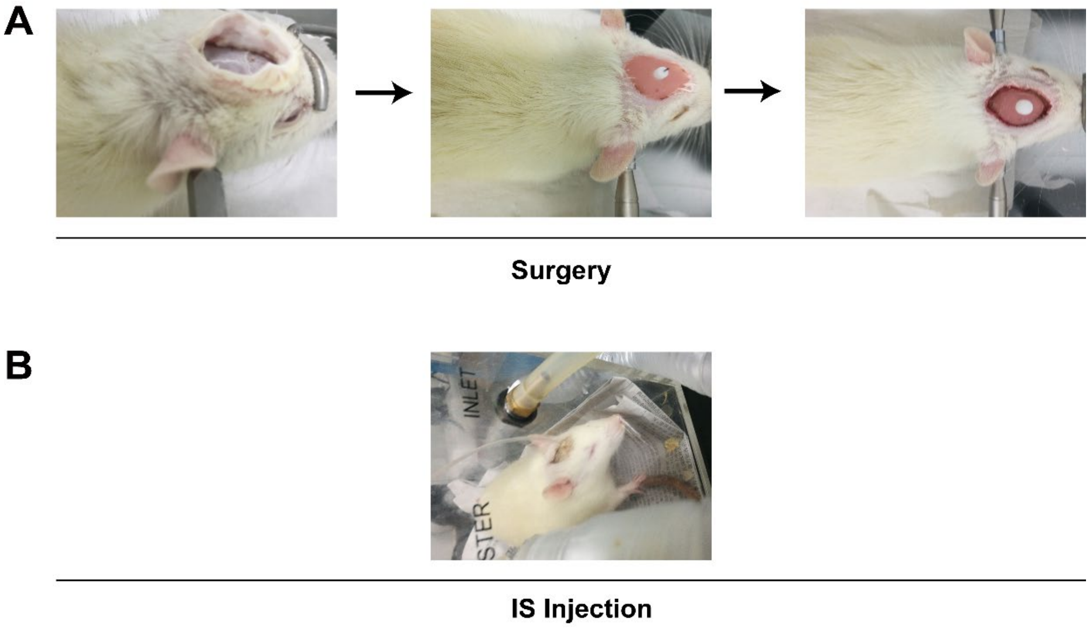

Supplementary Figure 1. Dural cannulation surgery **(A)** and IS-injection **(B)**.

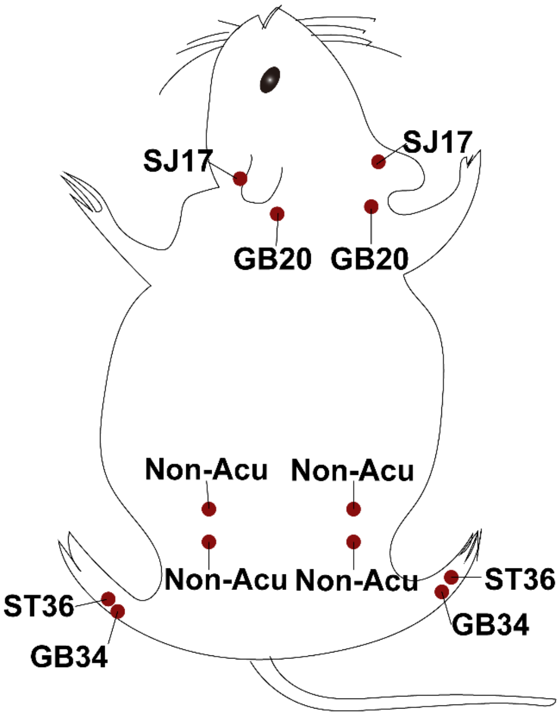

Supplementary Figure 2. The specific location of applied acupoints in rat.

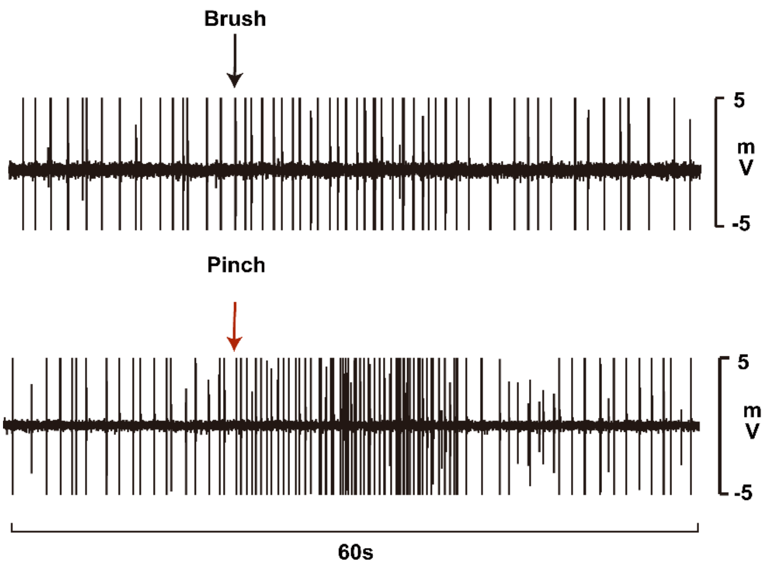

Supplementary Figure 3. WDR neurons were recognized based on their responses to mechanical non-noxious (brushing with a soft brush) and noxious (pinch with forceps) stimulations of the face in rats. WDR, Wide-dynamic range.

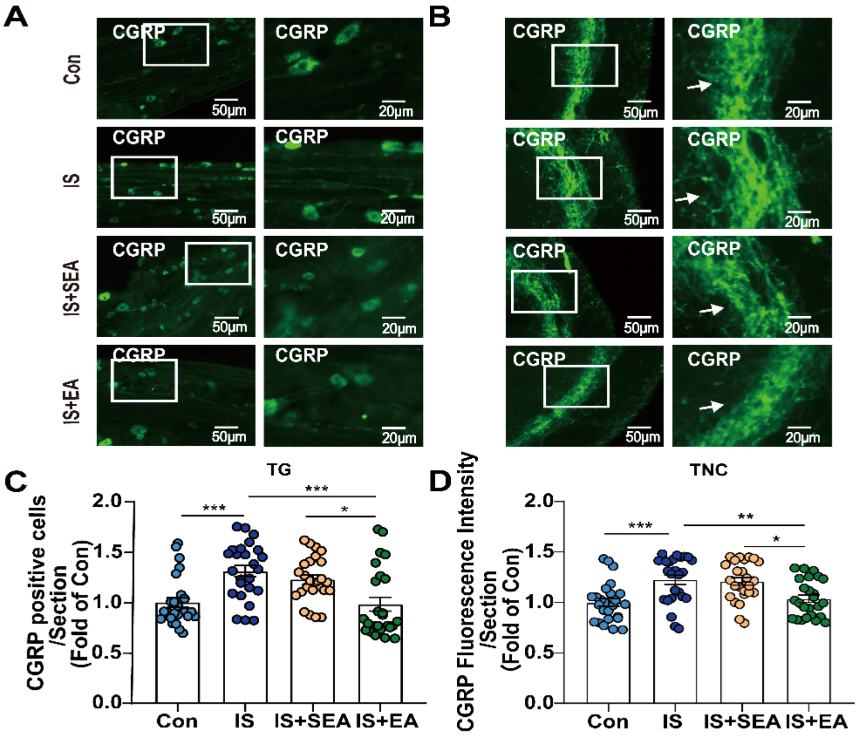

Supplementary Figure 4. Immunofluorescence results of CGRP in TG **(A and C)** and TNC **(B and D)** among the Con, IS, IS+SEA, IS+EA groups on day 8 after 4th IS injection. The green staining represents the CGRP, the white arrow points to the positive nerve fibers in TNC area. IS injection caused an increase in endogenous CGRP expression on the ipsilateral side in TG and TNC. Scale bars = 50μm. Quantitative analyses of CGRP to evaluate the numbers of positive cells **(C)** and the fluorescence intensity of positive nerve fibers **(D)** (n = 5, 5 sections/animal). One-way ANOVA followed by post hoc Tukey test. Group values are indicated by mean ± SEM. $^{*}P < 0.05$, $^{**}P < 0.01$, $^{***}P < 0.001$, $^{****}P < 0.0001$. CGRP, Calcitonin gene-related peptide; EA, electroacupuncture; SEA, sham electroacupuncture; IS, inflammatory soup.

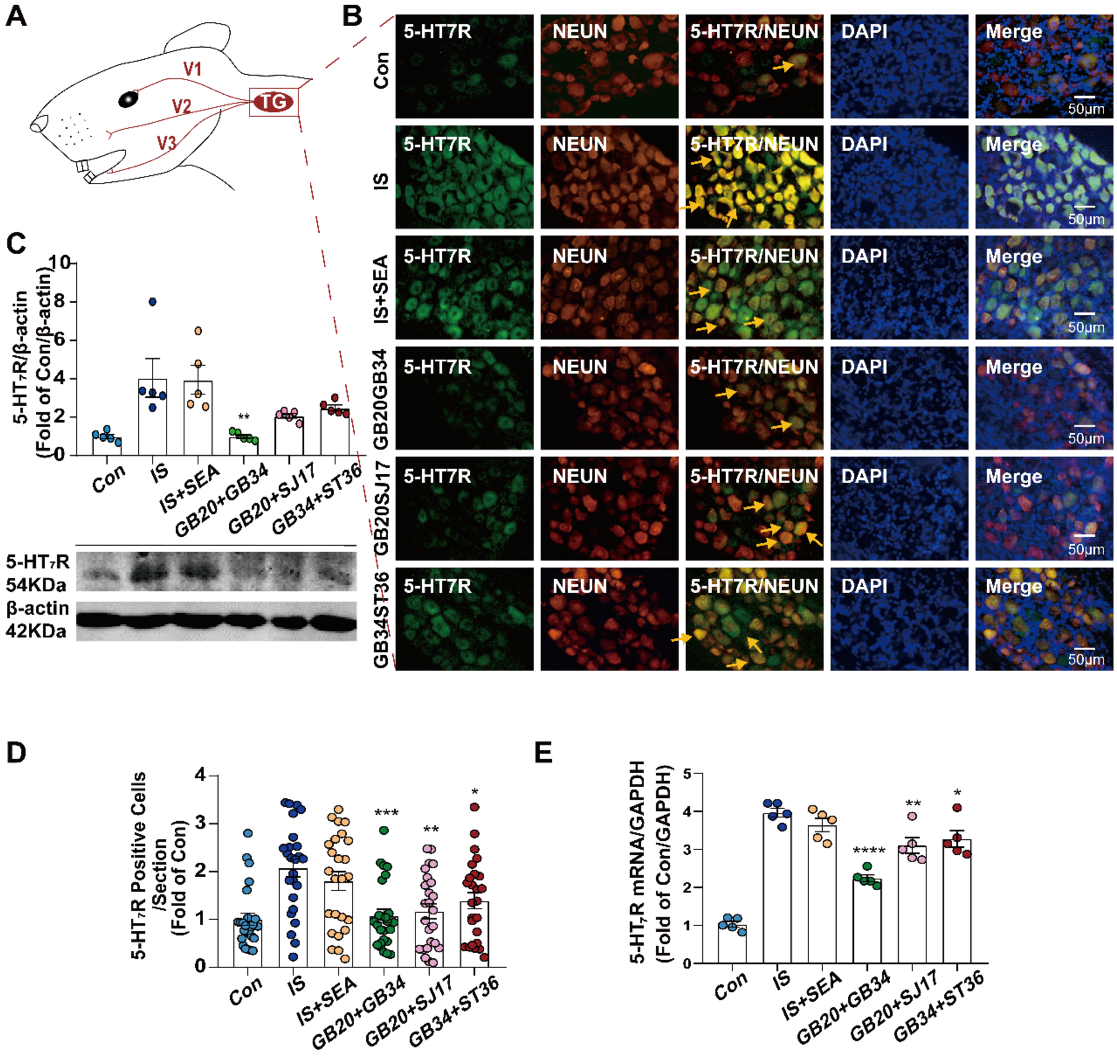

Supplementary Figure 5. Effects of EA on the formation of endogenous 5-HT$_7$R in the trigeminal ganglion (TG) on day 8 after 4th IS injection among Con, IS, IS+SEA, GB20+GB34, GB20+SJ17, GB34+ST36 groups. **(A)** Diagram of TG (red) in rat. **(B)** Immunohistochemical staining showed that 5-HT$_7$R (green) were expressed mostly in the individual neurons (NeuN, Neuron marker, red) of the TG in the indicated group. The orange arrow points to the positive cells in TG. Scale bars = 50μm. **(D)** The GB20+GB34 group showed the best effect that reduced in endogenous 5-HT$_7$R expression on the ipsilateral side in TG. Quantitative analyses of 5-HT$_7$R to evaluate the numbers of positive cells (n = 5, 5 sections/animal). **(C)** Representative western blot bands and quantitative analyses of 5-HT$_7$R in the six indicated groups. The same membranes were probed for 5-HT$_7$R with β-actin (n = 5). **(E)** The mRNA levels of 5-HT7R were also accessed by real-time polymerase chain reaction, and values were corrected by GAPDH in TG (n = 5). One-way ANOVA followed by post hoc Tukey test. Group values are indicated by mean ± SEM. $^{*}P < 0.05$, $^{**}P < 0.01$, $^{***}P < 0.001$, $^{****}P < 0.0001$. 5-HT$_7$R, 5-hydroxytryptamine (5-HT)$_7$ receptor; GAPDH, glycolytic enzyme glyceraldehyde 3-phosphate dehydrogenase; TG, trigeminal ganglion; SEA, sham electroacupuncture; EA, electroacupuncture; IS, inflammatory soup; GB20, Fengchi; GB34, Yanglingquan; SJ17, Yifeng; ST36, Zusanli.

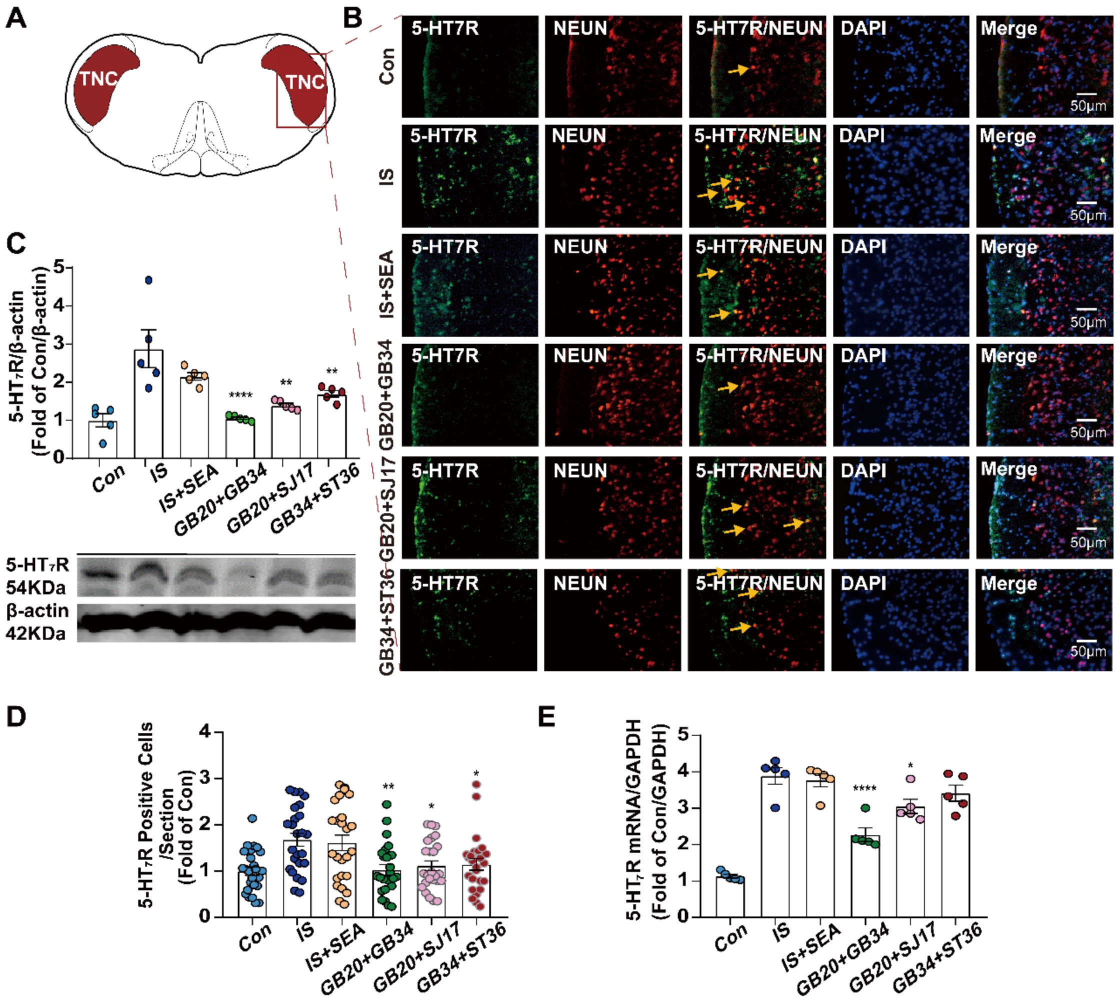

Supplementary Figure 6. Effects of EA on the formation of endogenous 5-HT$_7$R in the TNC on day 8 after 4th IS injection among Con, IS, IS+SEA, GB20+GB34, GB20+SJ17, GB34+ST36 groups. **(A)** Diagram of TNC (red) in rat. **(B)** Immunohistochemical staining showed that 5-HT$_7$R (green) were expressed mostly in the individual neurons (NeuN, Neuron marker, red) of the TNC among the Con, IS, IS+SEA, GB20+GB34, GB20+SJ17, GB34+ST36 groups. The orange arrow points to the positive cells in TNC. Scale bars = 50μm. **(D)** The GB20+GB34 group showed the best effect that reduced in endogenous 5-HT$_7$R expression on the ipsilateral side in TNC. Quantitative analyses of 5- HT$_7$R to evaluate the numbers of positive cells (n = 5, 5 sections/animal). **(C)** Representative western blot bands and quantitative analyses of 5-HT7R in the six indicated groups. The same membranes were probed for 5-HT7R with β-actin (n = 5). **(E)** The mRNA levels of 5-HT$_7$R were also accessed by real-time polymerase chain reaction, and values were corrected by GAPDH in TNC (n = 5). One-way ANOVA followed by post hoc Tukey test. Group values are indicated by mean ± SEM. $^{*}P < 0.05$, $^{**}P < 0.01$, $^{***}P < 0.001$, $^{****}P < 0.0001$. 5-HT$_7$R, 5-hydroxytryptamine (5-HT)$_7$ receptor; GAPDH, glycolytic enzyme glyceraldehyde 3-phosphate dehydrogenase; TNC, trigeminal nucleus caudalis; SEA, sham electroacupuncture; EA, electroacupuncture; IS, inflammatory soup; GB20, Fengchi; GB34, Yanglingquan; SJ17, Yifeng; ST36, Zusanli.
